# Bayesian Solutions for Assessing Differential Effects in Biomarker Positive and Negative Subgroups

**DOI:** 10.1002/pst.2456

**Published:** 2024-11-25

**Authors:** Dan Jackson, Fanni Zhang, Carl‐Fredrik Burman, Linda Sharples

**Affiliations:** ^1^ Statistical Innovation AstraZeneca Cambridge UK; ^2^ Statistical Innovation AstraZeneca Gaithersburg USA; ^3^ Statistical Innovation AstraZeneca Gothenburg Sweden; ^4^ Department of Medical Statistics London School of Hygiene and Tropical Medicine London UK

**Keywords:** informative prior distributions, medical decision making, prior sensitivity, subgroup analysis

## Abstract

The number of clinical trials that include a binary biomarker in design and analysis has risen due to the advent of personalised medicine. This presents challenges for medical decision makers because a drug may confer a stronger effect in the biomarker positive group, and so be approved either in this subgroup alone or in the all‐comer population. We develop and evaluate Bayesian methods that can be used to assess this. All our methods are based on the same statistical model for the observed data but we propose different prior specifications to express differing degrees of knowledge about the extent to which the treatment may be more effective in one subgroup than the other. We illustrate our methods using some real examples. We also show how our methodology is useful when designing trials where the size of the biomarker negative subgroup is to be determined. We conclude that our Bayesian framework is a natural tool for making decisions, for example, whether to recommend using the treatment in the biomarker negative subgroup where the treatment is less likely to be efficacious, or determining the number of biomarker positive and negative patients to include when designing a trial.

## 1 Introduction

Subgroup analyses are important in clinical trials because treatment efficacy may vary within a population. Here, our main focus lies in the situation where we have a binary biomarker that determines subgroup membership. Biomarkers may be prognostic (associated with the clinical outcome independently of treatment) or predictive (interact with treatment) [1]. Here, we focus on biomarkers that are plausibly predictive, so that the treatment efficacy is thought to differ for patients with different biomarker statuses. In general, biomarker measurements may be binary, ordinal or continuous, but here we assume they are binary. This is commonly the case, either naturally or after the dichotomisation of continuous biomarkers. Loss of information is associated with the simplifications gained by dichotomisation of continuous variables [2] but this remains a common and pragmatic way to include biomarkers in both statistical modelling and clinical decision‐making.

Clinical trials that include a binary biomarker, in design and analysis, are becoming more common due to the advent of personalised medicine. Other data structures can also be used to define two disjoint subgroups, such as age (adults and children [3]), disease severity [4] and cancer stage [5]. We will consider any such situation where efficacy may be larger in one subgroup than in the complement, for which our methods are equally applicable. As explained by Ballarini et al. [6], subgroup analyses are routinely performed in clinical trials to investigate whether treatment effects are homogeneous across the trial population. They illustrate a variety of visualisation techniques for this purpose. The International Council for Harmonisation (ICH) E17 guideline on *General principles for planning and design of Multi‐Regional Clinical Trials* [7] emphasises the importance of subgroup analyses, stating that in addition to investigating any regional or pooled subpopulation differences, other subgroup analyses will usually also be of interest. The European Medicines Agency (EMA) [8] *Guideline on the investigation of subgroups in confirmatory clinical trials* also emphasises the importance of subgroup analyses and accentuates the relevance of our methodology for the pharmaceutical industry.

The regulatory world of pharmaceutical statistics is dominated by the frequentist (or classical) approach. Here, a primary concern is whether estimated treatment effects achieve statistical significance, in each biomarker subgroup separately and also the trial population as a whole. Edgar et al. [1] focus on this type of problem. We, however, focus on Bayesian methods that are common in Health Technology Assessments following the regulatory phase of drug development and are a natural tool to aid medical decision‐making. This is because posterior distributions resulting from Bayesian analyses facilitate embedding statistical estimation within a probabilistic decision analysis. Although our Bayesian methodology is especially suitable for Health Technology Assessments, it also facilitates making decisions about the design of future trials where the size of the biomarker negative subgroup is to be determined, given the available prior information. We illustrate this using our second example below. These observations indicate that our methods may be most suitable at the design, and early phase, of clinical trial development, and also much later at the post regulatory phase. We return to this issue in the discussion.

There are several other potential benefits of using Bayesian methods [9], for example by including prior information precision may be increased so that uncertainty in medical decision‐making is reduced. Bayesian analyses may also be particularly valuable in situations where data are sparse, for example, because the disease or the event is rare, because relevant prior information can then be used to supplement analyses where little information would otherwise be available. Bayesian methods could also possibly be used for regulatory decisions on market authorisation, if regulatory authorities were willing to consider a range of reasonable priors, perhaps informed by previous trials or independent experts. Tidwell et al. [10] reviewed MD Anderson Cancer Center clinical trials and found that 283 out of 1020 (28%) had Bayesian components.

We extend the statistical model of Edgar et al. [1], by supplementing it with a variety of candidate prior distributions, and illustrate our methods using some examples. Our main aims are to complement their approach with Bayesian methods, to explore the use of a variety of informative prior distributions that may be motivated by other trial information, explicitly show concretely how relatively simple Bayesian methods can be useful in real applications and how they can also be useful for trial design. The use of informative priors for subgroup analysis and their application for trial design are especially novel features of our work. Sensitivity to prior distribution assumptions is a potential source of concern when performing Bayesian analyses [11]. The approach advocated here is to explore the implications of using different priors so that their impact can be assessed. Our proposals for prior distributions are by no means exhaustive, and we encourage the consideration of other possibilities. We provide computing codes that are intended to be easily modifiable for this purpose. Alternative Bayesian approaches for assessing treatment effect efficacy in subgroups include model averaging [12] and dynamic borrowing [13].

Our approach is also similar to that of Jones et al. [14], where we focus on the simpler situation where there are only two mutually exclusive subgroups. We instead explore the implications of a variety of different prior distributions for the difference in the treatment efficacy in these two patient groups. Prior distributions can be used to reflect our beliefs about the extent to which the treatment may be more effective in one subgroup, relative to the other. Edgar et al. [1] compared the conditional power of different testing rules for inferring efficacy in the biomarker negative subgroup given statistical significance in the entire study population. This is a crucially important consideration for the frequentist testing methods commonly used at the regulatory phase. The repeated sampling properties of Bayesian methods are also often of interest [15, 16, 17]. Exploration of the implications of sequential testing procedures using Bayesian models with different prior specifications could usefully extend the work of Edgar et al. [1]. Taking advantage of the sampling properties of Bayesian methods, we could quantify the consequences of different testing procedures, whilst taking into account our prior beliefs about the likely treatment effects in each subgroup. Such an investigation could be performed by restricting the MCMC iterations to those that are statistically significant, in a frequentist sense and in the entire population, and then calculating conditional powers as the proportions of iterations that also achieve this type of statistical significance in the biomarker negative population. In this paper, we use our Bayesian models to inform standard frequentist analyses in Section 5, where we consider designing a new trial. Alternatively, a fully Bayesian sequential testing approach might be possible, but we know from Lindley's paradox that Bayesian and frequentist approaches to hypothesis testing can give notably different results, depending on the prior distributions. A fully Bayesian extension of the conditional powers of Edgar et al. [1] would therefore be expected to be very sensitive to the prior specification because the additional challenges presented by subgroup analysis are likely to exacerbate the issues exemplified by Lindley's paradox.

The rest of the paper is set out as follows. In Section 2, we develop our Bayesian modelling framework, where we present both our model for the data and also some possibilities for prior distributions. In Section 3, we describe the numerical methods used and the targets for inference required for decision‐making. In Section 4, we illustrate our methods using two examples and we make some recommendations for those who may consider using our proposals for their subgroup analyses. In Section 5, we show how our methodology can be used at the trial design stage, where the size of each biomarker group is determined. We conclude in Section 6 with a discussion.

## 2 Modelling Framework

Following Edgar et al. [1], we assume that there are two mutually exclusive subgroups, B and C. We also define A=B∪C to be the entire study population. Edgar et al. used B− and B+ to denote the two subgroups, where B+ is the biomarker positive subgroup (for which the treatment is thought to be more effective). However, to allow more flexibility, we do not distinguish between the two subgroups in this way. Hence, we allow either subgroup B or C to be the biomarker positive subgroup.

We define the estimands of interest to be $\mu_{B}$ and $\mu_{C}$ in the two subgroup populations. For example, $\mu_{B}$ could be the log hazard ratio, log odds ratio or mean difference comparing the active treatment to the (active or placebo) control, in the B population. We assume that estimators of these effects are available from a new trial, either because they can be calculated using individual‐level data or because they can be ascertained from published information. We may also have informative historical data, or information available from subject matter experts, to provide informative prior distributions. We assume that, using a normal approximation,

(1) $$\left(\begin{array}{c} {\hat{\mu }}_{B}\\ {\hat{\mu }}_{C} \end{array}\right)|\left(\begin{array}{c} \mu_{B}\\ \mu_{C} \end{array}\right)∼N\left(\left(\begin{array}{c} \mu_{B}\\ \mu_{C} \end{array}\right),\left(\begin{array}{cc} \;\mathrm{Var}\;\left({\hat{\mu }}_{B}\right) & \;\mathrm{Cov}\;\left({\hat{\mu }}_{B},{\hat{\mu }}_{C}\right)\\ \;\mathrm{Cov}\;\left({\hat{\mu }}_{B},{\hat{\mu }}_{C}\right) & \;\mathrm{Var}\;\left({\hat{\mu }}_{C}\right) \end{array}\right)\right)$$

where the covariance matrix in Equation (1) is treated as fixed and known (estimates of these variance components can be used in this approximation provided that the trial is not small). In some instances ${\hat{\mu }}_{B}$ and ${\hat{\mu }}_{C}$ will be, conditionally on $\left(\mu_{B},\mu_{C}\right)$, independent so that $\;\mathrm{Cov}\;\left({\hat{\mu }}_{B},{\hat{\mu }}_{C}\right)=0$ in model (1). We can use the modelling framework of Edgar et al. [1] to justify model (1). A generalised linear model with linear predictor

(2) $$\eta_{i}=\beta_{0}+\beta_{1}T_{i}+\beta_{2}S_{i}+{\mathrm{\gamma X}}_{i}+\delta S_{i}T_{i}$$

is used to model the subgroup differences, for patient i, i=1,⋯N, where $T_{i}=0,1$ for control and active treatments, $S_{i}=0,1$ is an indicator for subgroup B and $X_{i}$ is a vector of additional baseline covariates. Then, $\mu_{B}=\beta_{1}+\delta$, and $\mu_{C}=\beta_{1}$, and analogous identities also apply to their estimators. The use of a normal approximation for these estimators then provides model (1). If no additional baseline covariates are included in the model, so that ${\mathrm{\gamma X}}_{i}=0$, then ${\hat{\mu }}_{B}$ and ${\hat{\mu }}_{C}$ are conditionally independent [1]. In the context of time‐to‐event data, ${\hat{\mu }}_{B}$ and ${\hat{\mu }}_{C}$ will be conditionally independent if survival modelling is performed on each subgroup separately because different patients then contribute to these two subgroup estimates. Hence, we adopt model (1) as our framework, with the understanding that in some instances it will also be possible to assume $\;\mathrm{Cov}\;\left({\hat{\mu }}_{B},{\hat{\mu }}_{C}\right)=0$. We explain why ${\hat{\mu }}_{B}$ and ${\hat{\mu }}_{C}$ are conditionally independent, if ${\mathrm{\gamma X}}_{i}=0$, in Appendix A. Other models could be used to obtain ${\hat{\mu }}_{B}$ and ${\hat{\mu }}_{C}$ and in our numerical examples, these estimates were taken from published results from Cox models. Bayesian methods could also be used to estimate subgroup treatment effects, and so obtain ${\hat{\mu }}_{B}$ and ${\hat{\mu }}_{C}$ and their covariance matrix, in model (1).

We use $\delta =\mu_{B}-\mu_{C}$ to describe the difference between the treatment effects in the two subgroups using a single parameter. This parameter is of direct inferential interest, for example, δ=0 means that the treatment is equally effective in the two subgroups (i.e. there is no treatment by subgroup interaction). If negative values of $\mu_{B}$ and $\mu_{C}$ indicate treatment benefit in the two subgroups, as is for the case in the examples in Section 4, δ<0 indicates that the treatment benefit is greatest in the B population.

We now have three linearly dependent parameters in our model $\mu_{B}$, $\mu_{C}$ and δ. However, we need only define a joint prior distribution for two of these parameters, because distributions (both prior and posterior) for the third are then obtained via the identity $\delta =\mu_{B}-\mu_{C}$. We propose some joint prior distributions for $\left(\mu_{C},\delta \right)$ immediately below. The use of a prior distribution for δ allows the possibility of shrinkage, where the subgroup‐specific effects $\mu_{B}$ and $\mu_{C}$ may be shrunk towards their mean. Our methods, and in particular when using vague priors, are closely related to standard Bayesian regression models. However, our implementations enable us to enforce $\delta =\mu_{B}-\mu_{C}$ in our modelling and are intended to be easily modifiable so that others can also use them. The close connection between our Bayesian methods, and those implemented in standard software, is an advantage of our proposals.

### 2.1 Prior Distributions

In most models we will use independent prior distributions for $\mu_{C}$ and δ. This keeps the modelling as simple as possible and invokes a prior association between $\mu_{C}$ and $\mu_{B}$, via the identity $\delta =\mu_{B}-\mu_{C}$, where $\;\mathrm{Cov}\;\left(\mu_{B},\mu_{C}\right)=\mathrm{Cov}\;\left(\mu_{C}+\delta ,\mu_{C}\right)=\mathrm{Var}\;\left(\mu_{C}\right)$. This positive prior association reflects the notion that if the treatment is beneficial in the B population, then it is also more likely to be beneficial in the C population. An alternative is to use independent prior distributions for $\mu_{B}$ and $\mu_{C}$, so that no shrinkage is conferred by the prior.

We will use the vague (weakly informative) prior $\mu_{C}∼N\left(0,100\right)$ in most analyses, where we assume that the prior variance of 100 is large compared to $\;\mathrm{Var}\;\left({\hat{\mu }}_{C}\right)$ in Equation (1). By using this vague prior we allow the likelihood to dominate, so that the vast majority of the information for $\mu_{C}$ will come from the data. In practice, we will often have prior knowledge about the likely treatment effect in one sub‐population, for example, from early phase trials or real‐world data, and we will illustrate this possibility in our first real example below. We will consider a variety of prior distributions for δ, some of which are highly informative, to incorporate prior knowledge about the difference in efficacy between the two groups.

#### 2.1.1 Normal Prior Distribution for δ

The simplest possibility is to assume the conjugate prior distribution $\delta ∼N\left(a,b^{2}\right)$, where a and b are fixed constants. This invokes a prior distribution of $\mu_{B}∼N\left(a,100+b^{2}\right)$ for the treatment effect in the B population. By taking a=0 and b=10, we use the same vague prior for δ that was proposed for $\mu_{C}$. If a=b=0 then we make the strong assumption of an equivalent treatment effect in populations B and C, because if δ=0, then $\mu_{B}=\mu_{C}$. Unless b=0, one consequence of assuming independent priors for $\mu_{C}$ and δ is that the prior variance of $\mu_{B}$ is then greater than the prior variance of $\mu_{C}$. This is of little concern in practice if sufficiently uninformative priors are used.

We can also consider a≠0, and other values of b, to explore the implications of using prior knowledge about the extent to which the treatment efficacy might be thought to be greater, or less, in the B population. For example, if negative $\mu_{B}$ and $\mu_{C}$ indicate treatment benefit in the two subgroups then we could take a<0 to reflect the prior belief that the treatment is more effective in the B population. We could also use smaller values of b to reflect our confidence in this. Another possibility is to truncate or censor δ to be the magnitude or sign thought plausible, but this is likely to be considered too informative in certain contexts. We will illustrate this idea using our two examples below. The posterior distributions of δ, $\mu_{B}$ and $\mu_{C}$ can be derived algebraically, as shown in Appendix B, and can be compared to their prior distributions to assess how the data have updated our prior beliefs.

#### 2.1.2 Discrete Prior Distribution for δ

It may be difficult to express prior beliefs as a continuous distribution. Another option is to assume a discrete distribution for the possible values of δ. Using a discrete distribution allows more flexibility in choice of shape of the distribution of beliefs. To implement this type of prior, we introduce a categorical random variable M. We assign a prior probability $p_{j}$ that M belongs to the *j*th category, j=1,⋯J, and we also assign a value $d_{j}$ to each category. We then define $\delta =d_{M}$, so that the implied prior distribution for δ is discrete, taking values $d_{j}$ with probability $p_{j}$. The $d_{j}$ and $p_{j}$ are fixed inputs to the analysis but δ is a random variable (because M is random, and $\delta =d_{M}$).

This type of prior is especially useful when eliciting informative prior distributions. For example, a discrete range of $d_{j}$ could be presented as candidate values of δ to subject matter experts. The experts then assign a probability $p_{j}$ to each $d_{j}$, reflecting their beliefs about the plausibility of these effects. Experts may find it easier to assign prior probabilities to a discrete set of $d_{j}$ in this way than attempt to specify an informative continuous prior for δ. By setting the $d_{j}$ to correspond to a representative quantity for a series of intervals, this type of prior can be elicited using the ‘chips and bins’ method. Bojke et al. [18] found that this was the preferred method of prior elicitation for 65% of 72 participants, compared to an alternative approach that relied on specifying the quartiles of a distribution.

The prior and posterior distributions of M can be compared to assess how the data have updated the prior probabilities for each $d_{j}$. Like its prior distribution, the posterior distribution of δ will be discrete, taking values $d_{j}$, j=1,⋯J. Alternatively, and as recommended by Bojke et al. [18], a discrete prior distribution could be used to motivate one that is continuous, resulting in a continuous posterior distribution.

#### 2.1.3 Spike and Slab Prior Distribution for δ

Another possibility that we consider is the spike and slab prior [19]. Here, we define the prior distributions for the spike $\delta_{0}∼N\left(0,0.0001\right)$ and the slab $\delta_{1}∼N\left(0,\tau^{2}\right)$, where $\tau^{2}>>0.0001$. If we defined $\delta =\delta_{0}$, then we would have a prior expressing the strong belief that the treatment effect is the same in the B and C populations. If we instead defined $\delta =\delta_{1}$ and τ=b we would have the normal prior in Section 2.1.1. A hyper‐prior on the probability that each prior model is ‘true’ completes the specification. The spike and slab prior therefore allows us to express uncertainty about our prior model choice.

To implement this type of prior, we introduce a random variable P that can take values on [0,1]; a uniform prior distribution for P is an especially simple choice and we use this throughout. We let P be the probability of an event for a Bernoulli random variable R, so that $P\left(R=1|P\right)=P$ and $P\left(R=0|P\right)=1-P$. We then define $\delta =\delta_{R}$, so that R=0 and R=1 indicate that the spike and slab priors are used for δ, respectively. The posterior distributions of R and P can be compared with their priors to determine how they are updated by the data, and so assess the strength of evidence for the spike versus the slab.

By directly allowing for the possibility of (almost) equal efficacy in the two subgroups via the spike, this prior can be expected to dilute the posterior evidence that δ≠0, compared to the vague prior in Section 2.1.1. This is because there will then be support for the spike, increasing the posterior density at δ=0 compared to less informative choices of prior distributions. The spike and slab prior may therefore be used to indicate the strength of the evidence for a subgroup difference, rather than incorporate prior information. For example, if the slab is found to be preferred to the spike then we can conclude that there is evidence for such a difference.

In Appendix C, we show, in a simplified setting where only the estimate $\hat{\delta }$ is used in analysis, that the posterior density of δ is a weighted average of two posterior distributions where the spike ($\delta ∼N\left(0,0.0001\right)$) and slab ($\delta ∼N\left(0,\tau^{2}\right)$) are used as the priors. We also show that the weight allocated to the slab tends towards zero as $\tau^{2}\to \infty$. It is therefore important to use a plausible value of $\tau^{2}$ in analysis and explore the sensitivity to this value. We illustrate this numerically for our two examples below.

#### 2.1.4 Joint Prior Distributions for $\mu_{B}$ and $\mu_{C}$

It may sometimes be convenient to instead specify prior distributions for $\mu_{B}$ and $\mu_{C}$, for example, when prior information is available and used to justify informative priors for these two parameters. This invokes a prior for $\delta =\mu_{B}-\mu_{C}$. An intuitively appealing joint vague prior is to assume that $\mu_{B}$ and $\mu_{C}$ are bivariate normal, centred at the origin, with the same large marginal variances (of 100, say) and with a correlation of 0.5. This implies the same vague marginal normal prior distributions for all three parameters $\mu_{B}$, $\mu_{C}$ and δ. Hence, this approach overcomes a potential objection to the methodology described in Section 2.1.1, where the prior variance of $\mu_{B}$ is greater than that of $\mu_{C}$. If all priors are intended to be vague, then they might reasonably be expected to have the same variance.

Another possibility is to use a discrete set of combinations of $\mu_{B}$ and $\mu_{C}$ to elicit informative prior distributions. For example, a discrete set of values of $\mu_{C}$ (say) could be presented to experts and a marginal prior distribution elicited. Then, a discrete set of values of $\mu_{B}$ could be presented and the conditional prior distribution of $\mu_{B}$, given each value of $\mu_{C}$, could be elicited. This provides an informative discrete joint prior distribution for $\mu_{B}$ and $\mu_{C}$, where the prior for δ is implied by the identity $\delta =\mu_{B}-\mu_{C}$. This discrete joint prior should in general be used to motivate a continuous bivariate prior. Determining which two of the three parameters to specify priors directly for is immaterial, provided that the same joint prior for $\left(\mu_{B},\mu_{C}\right)$ is used.

In Section 4.1.1, we will illustrate the use of a more complicated joint, correlated, prior for $\mu_{B}$ and $\mu_{C}$. This is intended to demonstrate how flexible our modelling can be.

## 3 Computation and Targets for Inference

We define the joint prior distribution for $\left(\mu_{C},\delta \right)$ using the methods described in Sections 2.1.1, 2.1.3, and the joint prior distribution for $\left(\mu_{B},\mu_{C}\right)$ when using the methods in Section 2.1.4, so that the prior for the third parameter is defined via the identity $\delta =\mu_{B}-\mu_{C}$. We then use standard Bayesian methods to update these prior distributions, via the likelihood from model (1), and so obtain the corresponding posterior distributions. The package R2jags was used to implement the Markov Chain Monte Carlo (MCMC) methods needed to evaluate these posterior distributions and WinBUGS [20] version 14 was used to double‐check the results. Two Markov chains with 50,000 iterations per chain (with a thinning of 2), and burn‐ins of 20,000 iterations, were used throughout. The likelihood from model (1) is implemented using a multivariate normal distribution; in situations where $\;\mathrm{Cov}\;\left({\hat{\mu }}_{B},{\hat{\mu }}_{C}\right)=0$ in model (1), this can be evaluated more simply using two independent implementations of the univariate normal distribution. As explained in Section 2.1.1, for the normal prior for δ, the posterior distributions of all three model parameters can be derived algebraically. For consistency across all priors, the same numerical methods were used throughout, but the algebraic results for the normal prior were used to check the corresponding numerical results.

Three main targets for inference are the posterior distributions of $\mu_{B}$, $\mu_{C}$ and δ, where δ is of primary interest because it quantifies the difference in the treatment efficacy in the two subgroups. In the examples that follow, these will be summarised using posterior means, standard deviations and credible intervals within this Bayesian framework. Further quantities are also potentially of inferential interest. For example, by conceptualising the entire study population A, where the proportion π are B patients, the posterior distribution of

(3) $$\mu_{A}={\pi \mu }_{B}+\left(1-\pi \right)\mu_{C}$$

could be another useful aid for decision‐making. This type of estimand may be of particular interest when the sampling proportion for the trial π differs in another target population. To target another population we simply replace π in Equation (3) with the required proportion of B patients. The estimand $\mu_{A}$, interpreted as the treatment effect in population A, will be exact for some outcomes, for example, unadjusted mean differences. However, Equation (3) is only an approximation for examples that like ours model estimated log hazard ratios. We propose using Equation (3) because it is so broadly applicable and simple to implement. However, for some types of outcome, alternative overall or pooled estimands combining those from subgroups B and C populations are available. For example, for binary outcome data, pooling using Cochran–Mantel–Haenszel methods might be preferred. The relationship between the treatment effects in Equation (3) only holds exactly for collapsible effect sizes. We return to this issue in the discussion.

Posterior distributions of other functions of $\mu_{B}$, $\mu_{C}$ and δ are evaluated by defining the quantity of interest and summarising the corresponding MCMC output. For example, indicators for $\mu_{B}$, $\mu_{C}$ and δ being positive or negative could be defined, so that the posterior probability that the treatment is effective in each population, and also the posterior probability that the treatment is more effective in the B population, may be calculated. We will introduce several further quantities of inferential interest in Section 5, where we examine how our methods may be used when designing a trial.

## 4 Examples

### 4.1 Example 1: The STAMPEDE Trial

Eligible patients for STAMPEDE had prostate cancer that was newly diagnosed and metastatic, node‐positive or high‐risk locally advanced. The aim was to test whether the addition of further treatments (abiraterone and prednisolone) to androgen‐deprivation therapy (ADT) improves overall survival (OS) if used in the first‐line setting [5]. A total of 1917 patients underwent randomisation, of whom 1002 (52%) had metastatic disease, and key subgroup analyses according to metastatic status were pre‐specified [5]. The OS subgroup results re‐examined here are shown in Figure 2A of James et al. [5], where estimated hazard ratios (and 95% confidence intervals) of 0.75 (0.48, 1.18) and 0.61 (0.49, 0.75) are reported for the non‐metastatic (subgroup B) and metastatic (subgroup C) patients, respectively (both favouring combination therapy compared to ADT alone). A *p*‐value for the interaction (evidence of a subgroup effect) of 0.37 is also reported [5]. Although there is not a statistically significant difference between the treatment effects in the two subgroups, the result for the metastatic subgroup is statistically significant at conventional thresholds whereas those for the non‐metastatic subgroup are not.

We use this information to explore this subgroup analysis from a Bayesian perspective. We compute estimated log hazard ratios for the two subgroups, ${\hat{\mu }}_{B}=\log\left(0.75\right)\approx -0.288$ and ${\hat{\mu }}_{C}=\log\left(0.61\right)\approx -0.494$, and their corresponding standard errors (whose squared values are $\;\mathrm{Var}\;\left({\hat{\mu }}_{B}\right)$ and $\;\mathrm{Var}\;\left({\hat{\mu }}_{C}\right)$ in model 1), as the ratio of the length of the confidence intervals on the logarithm scale and 2×1.96. We assume that ${\hat{\mu }}_{B}$ and ${\hat{\mu }}_{C}$ are conditionally independent, so that in model (1) we have $\;\mathrm{Cov}\;\left({\hat{\mu }}_{B},{\hat{\mu }}_{C}\right)=0$. Note that this does not imply that the posterior correlation between $\mu_{B}$ and $\mu_{C}$ is zero.

We apply our vague normal prior distribution for δ with a=0 and b=10 where we also use the vague prior $\mu_{C}∼N\left(0,100\right)$ (Section 2.1.1). In the web Supporting Information, we apply our discrete prior for δ (Section 2.1.2) with $d_{1}$ = −2.0, $d_{2}$ = −1.9, $d_{3}$ = −1.8, ⋯, $d_{41}$ = 2.0 and $p_{j}=1/41$ for j=1,2,⋯,41. This discrete prior places equal prior probability on each possible value δ that are symmetric around 0, so this prior is also not intended to be informative within the range $\left[-2,2\right]$. These results and codes are provided so that others may adapt them for use with an informative discrete prior for δ, for example, one elicited from experts.

We perform three types of analyses that use informative priors. Firstly, we apply the spike and slab prior as explained in Section 2.1.3, with three different values of τ=10, 1, 0.3. The first of these τ corresponds to using a vague prior for the slab, and the second is not overly informative because it considers large values of δ to be plausible. The final τ=0.3 instead considers only more moderate values of δ to be plausible. Note that δ is the difference between log hazard ratios in the two subgroups, so that $\exp\left(\delta \right)$ is a ratio of hazard ratios. Over 95% of probability for this parameter in the slab prior lies in the interval (−0.6, 0.6), so that ratios of hazard ratios outside the interval (0.5, 2), say, are not given much support in this prior specification.

Secondly, we apply our vague normal prior distribution for δ (Section 2.1.1) but for $\mu_{C}$ we apply informative prior distributions motivated by Ryan et al. [21]. Their trial concerned patients with metastatic prostate cancer (and hence we used this to inform $\mu_{C}$). Here a hazard ratio for OS of 0.75, with a 95% confidence interval of (0.61, 0.93), is reported that compares patients receiving abiraterone and prednisone compared to those who receive placebo plus prednisone. Although there are differences in the treatment regimens, a key similarity is that abiraterone is the main active treatment in both trials, motivating the use of information from Ryan et al. [21] in the prior specification. Transforming the point estimate of 0.75 and corresponding 95% confidence interval to the log hazard ratio scale, we obtain the informative prior $\mu_{C}∼N\left(-{0.288, 0.108}^{2}\right)$. Here, the prior variance of $\mu_{C}$ was calculated using the 95% confidence interval of (0.61, 0.93) and the same approach as when computing the variances of ${\hat{\mu }}_{B}$ and ${\hat{\mu }}_{C}$. However, as explained by Lunn et al. [22], by using this prior directly we essentially pool the information from Ryan et al. [21] with our data in the form of a meta‐analysis. We may be reluctant to do this, because we may not consider data from other sources to have the same relevance. We, therefore, follow a suggestion in Section 5.3.2 of Lunn et al. [22] by using ‘power priors’ [23] to discount the prior information from Ryan et al. [21]. More specifically, we also use priors of $\mu_{C}∼N\left(-{0.288, 0.108}^{2}/k\right)$, where k=0.75, 0.5, 0.25. As the value of $k\in \left(0,1\right)$ decreases, we further down‐weight the prior information from Ryan et al. [21], and so move from a very informative prior for $\mu_{C}$ to a vague prior. Alternative prior specifications are available for this purpose [24] but power priors are especially simple and direct. As a third type of informative prior, we implemented our vague normal priors for $\mu_{C}$ and δ where the prior $\mu_{C}$ was truncated to be less than −0.23, so that only hazard ratios less than 0.8 in subgroup C are considered plausible.

Finally, we also apply the vague bivariate normal distribution for $\mu_{B}$ and $\mu_{C}$ (Section 2.1.4). This analysis overcomes the concern that our choice of vague priors in Section 2.1.1 implies a larger prior variance for $\mu_{B}$ than $\mu_{C}$. This is unlikely to substantially alter the results.

**FIGURE 1:**
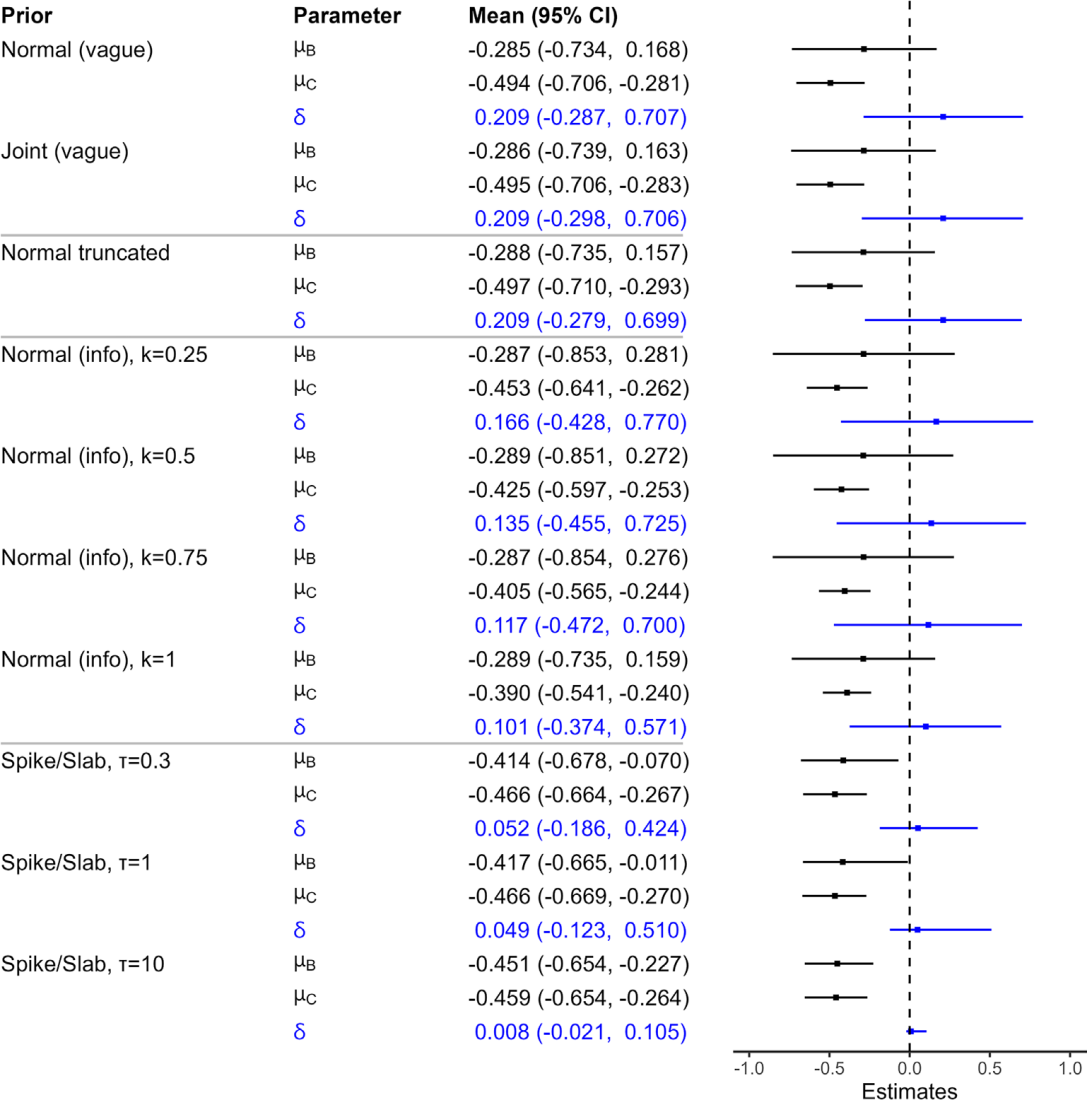
Posterior means and credible intervals for Example 1 (Section 4.1). ‘Normal (vague)’ and ‘Joint (vague)’ are results using vague priors, Sections 2.1.1 and 2.1.4, respectively. ‘Normal truncated’ are results as in ‘Normal (vague)’ but where $\mu_{C}$ is truncated to be less than −0.23. ‘Normal (info)’ are results using the informative normal prior $\mu_{C}∼N\left(-{0.288, 0.108}^{2}/k\right)$ with the value of k indicated; as k increases this prior becomes more informative. Finally, ‘Spike/Slab’ are results using the spike and slab prior, where τ is the standard deviation used for the slab.

The posterior distributions of $\mu_{B}$, $\mu_{C}$ and δ are summarised in Figure 1. The results in Figure 1 are split into four sections, depending on whether a vague prior, the truncated prior for $\mu_{C}$, an informative power prior, or the spike and slab prior was used. The parameter $\delta =\mu_{B}-\mu_{C}$ represents the additional increase in the log hazard ratio due to treatment in the B population compared to the C population (all else being equal); δ>0 therefore indicates that the treatment effect is greater in the C population. The results for the vague priors are in good agreement with the frequentist point estimates and confidence intervals [5], as expected because these priors are not very informative. The results for the spike and slab prior distribution are sensitive to the value of τ used [25] and for the large τ=10 the spike appears to dominate the slab as expected (see Section 2.1.3 and Appendix C). However the point estimates for moderate values of τ=1 and τ=0.3 are more similar, and together these results show the lack of evidence of a difference in treatment effect in the two subgroups from a Bayesian perspective. Comparing the results using the informative prior distribution for $\mu_{C}$, to those using the vague normal prior in Table 1, we can see that this informative prior has considerable impact on $\mu_{C}$. This has direct consequences for the posterior mean of δ, roughly halving its value. However, the two posterior distributions of $\mu_{B}$ are very similar. This is because the informative prior introduces information relating to $\mu_{C}$, rather than $\mu_{B}$. As we decrease k, and so use the power prior to increasingly discount the prior information, the results become more similar to those using vague priors, as expected. The truncation of $\mu_{C}$ to be less than −0.23 has little impact because there is not a great deal of support for $\mu_{C}$ to be greater than this.

**TABLE 1:** Joint discrete prior for $\mu_{B}$ and $\mu_{C}$ in Example 1.

	$\mu_{B}=\log\left(0.5\right)$	$\mu_{B}=\log\left(0.6\right)$	$\mu_{B}=\log\left(0.7\right)$	$\mu_{B}=\log\left(0.8\right)$	$\mu_{B}=\log\left(0.9\right)$	$\mu_{B}=\log\left(1.0\right)$
$\mu_{C}=\log\left(0.5\right)$	0.0030	0.0025	0.0020	0.0015	0.0005	0.0005
$\mu_{C}=\log\left(0.6\right)$	0.0090	0.0315	0.0225	0.0135	0.0090	0.0045
$\mu_{C}=\log\left(0.7\right)$	0.0150	0.0300	0.1050	0.0900	0.0450	0.0150
$\mu_{C}=\log\left(0.8\right)$	0.0000	0.0175	0.0525	0.1400	0.1050	0.0350
$\mu_{C}=\log\left(0.9\right)$	0.0000	0.0000	0.0100	0.0400	0.0900	0.0600
$\mu_{C}=\log\left(1.0\right)$	0.0000	0.0000	0.0005	0.0020	0.0050	0.0425

Collectively, the results in Figure 1 summarise the conclusions across a wide range of prior specifications. The overall impression is that there is strong evidence of a treatment effect in population C. However, the evidence is, at best, much weaker in population B. Despite this, there is no clear evidence of a difference between the treatment effects in the two patient populations. More information about the treatment efficacy in population B, and its difference across the two populations, would be valuable.

#### 4.1.1 A Correlated Joint Prior Distribution for $\mu_{B}$ and $\mu_{C}$

We now demonstrate how an informative bivariate normal distribution could be derived using expert elicitation. Thereafter, we extend this process to derive a novel bivariate prior that accommodates additional concerns that might be expressed by clinicians.

To elicit a bivariate prior density for $\left(\mu_{B},\mu_{C}\right)$, we first specify a range of plausible effects sizes in population C, in this case, hazard ratios of 0.5, 0.6, 0.7, 0.8, 0.9 and 1. Prior probabilities of the six values of $\mu_{C}$, the corresponding log hazard ratios shown in Table 1, are then elicited from clinicians. Conditional on each value of $\mu_{C}$, prior probabilities of the same six values of $\mu_{B}$ can then be elicited. For example, to obtain conditional prior probabilities of $\mu_{B}$ given $\mu_{C}=\log\left(1\right)=0$, we ask ‘If you knew that the drug truly has no efficacy in population C, what value would you give to the probability that the hazard ratio in population B is 0.5, 0.6, 0.7, 0.8, 0.9 or 1?’ Having determined suitable prior probabilities $P\left(\mu_{C}\right)$, and $P\left(\mu_{B}|\mu_{C}\right)$, the joint prior probabilities $P\left(\mu_{B},\mu_{C}\right)$ shown in Table 1 were obtained by taking their product. The joint prior distribution in Table 1 was not formally elicited, but is based on informal discussions with clinicians and is used for illustrative purposes. The six discrete values of $\mu_{B}$ and $\mu_{C}$ were chosen to simplify the elicitation of prior distributions, as in the ‘chips and bins’ method [18] (Section 2.1.2). Holzhauer et al. [26] describe the SHELF extension method, in Section 3.3, that similarly elicits a marginal distribution for one variable and then a conditional distribution for a second variable, given the first.

In fact, clinicians' beliefs concerning these parameters were continuous and we start by estimating a bivariate normal prior density for $\left(\mu_{B},\mu_{C}\right)$ that is consistent with this grid of joint probabilities, that is, that has the mean and covariance structure implied by Table 1. This prior resulted in a posterior means of −0.310, −0.402 and 0.092, with credible intervals of (−0.552, −0.063), (−0.564, −0.238) and (−0.151, 0.338), for $\mu_{B}$, $\mu_{C}$ and δ, respectively.

Suppose that clinicians were keen to explicitly represent the following concerns: harms due to treatment are considered unlikely (i.e. $\mu_{B}>0$ and $\mu_{C}>0$ are implausible), there is a non‐negligible probability that there is no treatment effect in either population (i.e. a point mass at $\left(\mu_{B},\mu_{C}\right)=\left(0,0\right)$ is considered plausible) and the mean and variance of the conditional prior for $\mu_{B}$ could depend on $\mu_{C}$. To capture these concerns, we based our prior specification on a ‘right‐rectified normal distribution’. This distribution was easily implemented in Bayesian software by initially defining $Y∼N\left(\mu ,\sigma^{2}\right)$ as a normal random variable, and then the right‐rectified normal distribution, $Z∼\mathrm{RN}\left(\mu ,\sigma^{2}\right)$, as min $\left(0,Y\right)$. The right‐rectified normal distribution is equivalent to a normal distribution except that positive values are reset to zero.

Our novel prior has marginal distribution

(4) $$\mu_{C}∼\mathrm{RN}\left(a,b^{2}\right),$$

where a and b>0 are fixed constants, analogous to those for the bivariate normal. This prior distribution allows a point mass at $\mu_{C}=0$ of $1-\Phi \left(\left(-a/b\right)\right)$, where $\Phi \left(\cdot \right)$ is the standard normal cumulative distribution function, reflecting the possibility that the treatment is not effective in population C. For the conditional prior distribution $\mu_{B}|\mu_{C}$, we adopted a prior distribution of the form

(5) $$\mu_{B}|\mu_{C}∼\mathrm{RN}\left(c\mu_{C},\max\;\left(d^{2}+e\mu_{C},0\right)\right)$$

where c, d>0 and e are further fixed constants to be determined. We can then obtain the joint prior distribution of $\mu_{B}$ and $\mu_{C}$ as the product of the prior distributions in Equations (4) and (5). This novel prior specification is highly unusual but is closely related to a bivariate normal; if we instead use normal distributions in Equations (4) and (5), include an intercept in the mean of $\mu_{B}|\mu_{C}$ and set e=0 in Equation (5), then this more conventional prior distribution is obtained.

Full details are provided in the codes in the web Supporting Information. Briefly, we define the set of six intervals on the real line.

$$\begin{array}{c} \begin{array}{l} S=\{(-\infty ,\log\left(0.55\right)],(\log\left(0.55\right),\log\left(0.65\right)],(\log\left(0.65\right),\log\left(0.75\right)],\\ \;(\log\left(0.75\right),\log\left(0.85\right)],\left(\log\left(0.85\right),\log\left(0.95\right)\right],\left(\log\left(0.95\right),\infty \right)\} \end{array} \end{array}$$

and we take the Cartesian product S×S to split the $\mu_{B},\mu_{C}$ plane into 36 rectangular regions. We then calculated, approximately, the prior probability that $\left(\mu_{B},\mu_{C}\right)$ lies in each of these 36 regions. This was achieved by computing the products of the prior probabilities that $\mu_{C}$ lies in each member of s∈S from model (4), and the conditional probability that $\mu_{B}$ lies in each s given $\mu_{C}$ from model (5), where the values of $\mu_{C}$ in Table 1 that lie within each s were used to approximate these conditional probabilities. The resulting probabilities that $\left(\mu_{B},\mu_{C}\right)$ lie in each of the 36 regions are functions of a, b, c, d and e and were interpreted as expected ($E_{i}$) probabilities under the prior specification. The probabilities shown in Table 1 are the corresponding observed probabilities ($O_{i}$). We numerically minimised the sum of squared residuals, $\sum_{i}{\left(O_{i}-E_{i}\right)}^{2}$, to determine suitable values for the five fixed constants to use in analysis.

**FIGURE 2:**
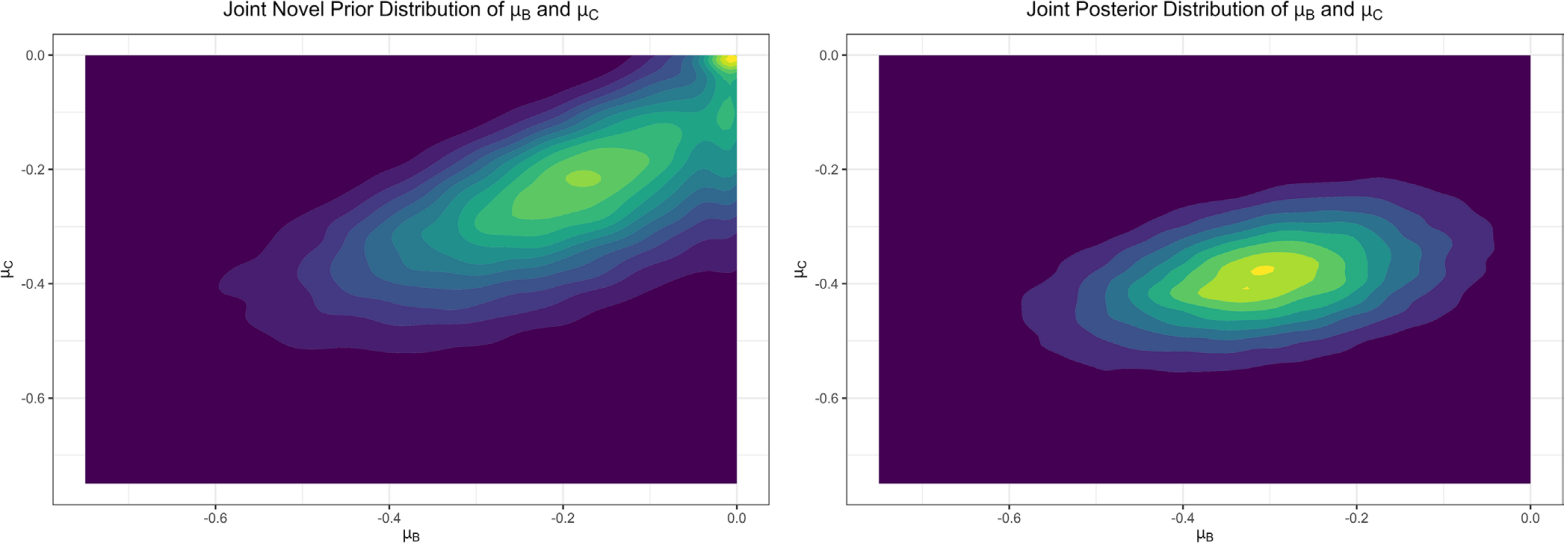
The joint prior (left) and posterior (right) distribution of $\left(\mu_{B},\mu_{C}\right)$ using a novel prior based on the right rectified normal distribution (Section 4.1.1), obtained by applying kernel density estimation to the MCMC iterations.

This resulted in a=−0.252, b=0.131, c=0.816, d=0.054 and e=−0.045. Using this prior specification provided posterior means of −0.307, −0.391 and 0.084, with credible intervals of (−0.570, −0.056), (−0.552, −0.232) and (−0.159, 0.336), for $\mu_{B}$, $\mu_{C}$ and δ, respectively. In Figure 2, we show the joint prior and posterior distribution of $\left({\hat{\mu }}_{B},{\hat{\mu }}_{C}\right)$, where these distributions were obtained by applying kernel density estimation to the MCMC iterations. The joint prior distribution was calculated by refraining from providing the estimated subgroup treatment effects to update it. The main implications of using this prior distribution, compared to a vague prior, are that we now infer a smaller treatment effect in population C and a smaller difference δ between the treatment effects in the two patient populations. However, the most important observation is that our approach can readily accommodate bespoke and non‐standard prior specifications. We return to this issue in the discussion.

### 4.2 Example 2: The METEOR Trial

The METEOR trial [27] evaluated the effect of Cabozantinib compared to Everolimus on progression‐free and OS in patients with advanced or metastatic renal cell cancer (RCC) that has progressed after prior VEGFR tyrosine kinase inhibitor therapy. Given that it is known that bone metastases are associated with increased morbidity in patients with RCC, efficacy and safety were analysed for subgroups defined by bone metastasis status at baseline. Of all the 658 patients randomly assigned, 142 (22%) belonged to a pre‐specified subgroup having bone metastasis.

Escudier et al. [27] provide the OS by subgroup results in their Figure 3, where the estimated hazard ratio is reported as 0.54 (95% confidence interval, 0.34–0.84) in patients with bone metastases (subgroup B) and 0.71 (95% confidence interval, 0.55–0.91) in those without bone metastases (subgroup C). After log transformation, ${\hat{\mu }}_{B}=\log\left(0.54\right)\approx -0.616$ and ${\hat{\mu }}_{C}=\log\left(0.71\right)\approx -0.342$. We apply our Bayesian approach to re‐evaluate the treatment effects for both subgroups in the way described in more detail for our first example in Section 4.1 when implementing the two vague, and spike and slab, and ‘truncated $\mu_{C}$′ priors.

**FIGURE 3:**
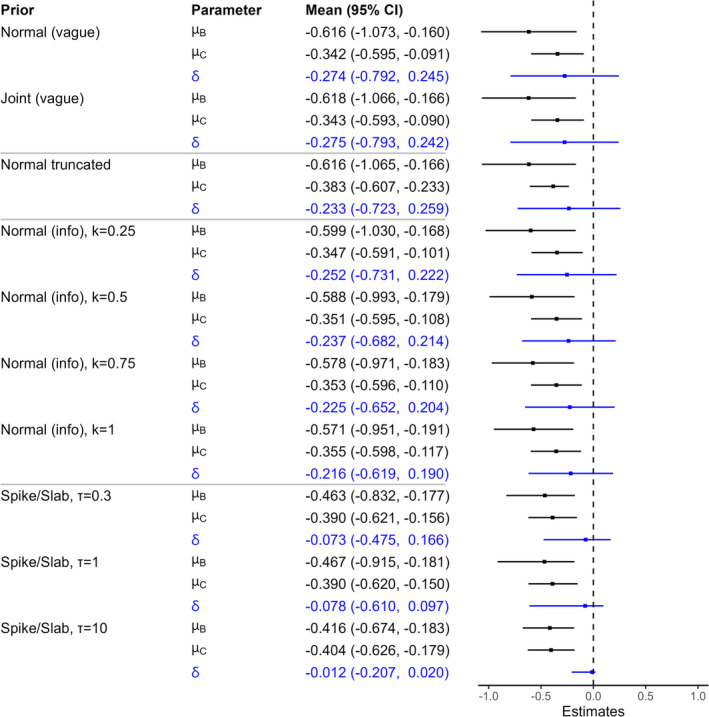
Posterior means and credible intervals for Example 2 (Section 4.2). ‘Normal (vague)’ and ‘Joint (vague)’ are results using vague priors, Sections 2.1.1 and 2.1.4, respectively. ‘Normal truncated’ are results as in ‘Normal (vague)’ but where $\mu_{C}$ is truncated to be less than −0.23. ‘Normal (info)’ are results using the informative normal prior $\delta ∼N\left(-{0.122,0.334}^{2}/k\right)$ with the value of k indicated; as k increases this prior becomes more informative. Finally, ‘Spike/Slab’ are results using the spike and slab prior, where τ is the standard deviation used for the slab.

We also use an informative prior distribution for $\delta =\mu_{B}-\mu_{C}$ derived from Choueiri et al. [28]. As in the first example there are differences in the treatment regimens, but a key similarity is that cabozantinib is an active treatment in both trials. Choueiri et al. [28] showed that Nivolumab plus Cabozantinib had significant benefits over sunitinib with respect to progression‐free survival and OS in both subgroups (with and without bone metastases) of patients with RCC. The OS hazard ratios are 0.54 (95% confidence interval, 0.32–0.92) for patients with bone metastases (B) and 0.61 (95% confidence interval, 0.41–0.89) for patients without bone metastases (C). The prior distribution $\delta ∼N\left(-{0.122,0.334}^{2}\right)$ was obtained by taking the difference between these two estimated hazard ratios on the log scale and assuming that they are independent. As in the first example, we also use ‘power priors’, of the form $\delta ∼N\left(-{0.122,0.334}^{2}/k\right)$ with k=0.75, 0.5, 0.25, to down‐weight this prior information.

We summarise the posterior distributions of $\mu_{B}$, $\mu_{C}$ and δ in Figure 3. The results for the vague priors are in good agreement with the frequentist point estimates and confidence intervals [27], as expected. Comparing the results using the informative prior for δ to those using the vague prior, we can see that this informative prior has considerable impact on δ. The posterior mean of δ lies between the mean of its informative prior distribution and its posterior mean when instead using vague priors. The posterior means of $\mu_{B}$ and $\mu_{C}$ are both affected by the informative prior for δ, but this is greatest for $\mu_{B}$ because there is less data available for this subgroup. The truncation of $\mu_{C}$ to be less than −0.23 has little impact, for similar reasons as in the first example. The observations for the spike and slab prior and the power priors are also similar to those from the first example.

The overall impression from Figure 3 is that there is evidence of a treatment effect in both patient populations. There is no evidence that the treatment effect is greater in population B. However, further information would be valuable in order to estimate the difference between the subgroup treatment effects with greater precision.

### 4.3 Recommendations

We have implemented two different ways to specify vague (weakly informative) prior distributions. We propose always beginning with an analysis that uses this type of prior, where we should check that parameter estimates are consistent with frequentist results.

The possibility of exploring the impact of using external information should then be considered, where informative normal distributions are a convenient way to incorporate this additional information. Methods that discount the influence of candidate informative prior distributions should be used, for example, we have used power priors for this purpose. This enables us to understand the implications of making use of external information but treating it as less relevant. Further modifications can be easily incorporated with minor changes to the MCMC code. For example, priors that are truncated, multimodal or allow for other complex dependencies can be included to reflect clinicians' concerns or opinions.

In practice, a joint prior distribution can be difficult to elicit. We recommend using established prior elicitation methods [18, 26] to elicit a marginal prior for treatment effect in the patient group with the most established information base. A prior distribution for the treatment effect in other patient group, or the relative difference between the treatment effects in the two groups, can then be elicited conditional on this marginal prior. Alternatively, other methods for allowing for associations between the marginal prior distributions may be used [26]. Although it may be useful to use a discrete prior for elicitation, the resulting joint prior should be transformed into a continuous joint prior to ensure continuous posterior distributions and results from discrete priors could be used to help us understand the implications of this transformation. Defending informative prior distributions will be especially important in situations where they provide qualitatively different results to vague priors. This defence may prove challenging if the analysis is criticised by stakeholders who do not find the informative priors plausible.

We recommend that the spike and slab prior distribution should rarely be used for estimation. Only under exceptional circumstances, for example, when there is a strong prior belief that the treatment effect could be identical across the two populations, might this prior distribution be used for this purpose. It is, at best, very difficult to provide a concrete example of such a situation. Furthermore, the variance of the slab would need to be very judiciously selected and results can be expected to be sensitive to this.

## 5 Designing a New Trial

Bayesian methods may be especially useful in the context of designing a new trial, where the numbers of recruited biomarker positive and negative patients are to be determined. To illustrate this we explore the case where a trial similar to that of Escudier et al. [27], our second example above, is to be performed. A key aspect of trial design is determining the probability of trial success (achieving statistical significance with the intended directional effect). We extend our Bayesian modelling from Section 4.2 where the informative prior $\delta ∼N\left(-{0.122, 0.334}^{2}\right)$ was applied to the Escudier et al. [27] data. The approach adopted below allows us to directly, and explicitly, use prior information when designing a trial. It also allows us to compute posterior predictive distributions. However, it is only intended to be illustrative and we leave the assessment of its performance, relative to other methods and ideas, as further work. It is not essential to use information from another trial but if such information is available, it can be valuable in helping us make well informed decisions.

For illustrative purposes, suppose that such a trial with 900 patients and 1:1 randomisation is proposed where, like Escudier et al. [27], the concurrent evaluation of patients with and without bone metastases is to be performed. We suppose that follow‐up is intended to be for 2 years, the outcome of interest is OS, and there is doubt concerning whether to recruit 300 (1/3 of patients), 450 (1/2 of patients) or 600 (2/3 of patients) from subgroup B (with bone metastases). We therefore wish to better understand the implications of this decision. To apply our methods to address this, further information from Escudier et al. [27] will be used. From Figure 3 of Escudier et al., we expect a 60% event rate within the study period in the active arm for population B and a 80% event rate in the corresponding control arm. Similarly, we expect a 50% event rate in the active arm for population C and a 70% event rate in control group C. We predict the number of events (deaths) in each biomarker and treatment group combination as the products of patient numbers and event rates.

An example of this prediction, suppose that we decide to recruit 600 (2/3 of patients) from subgroup B. We then predict 600×1/2×0.6+600×1/2×0.8=420 events in the corresponding subgroup analysis, where the halves in these products are due to 1:1 randomisation between treatments within each subgroup (i.e. stratified randomisation). Similarly, we predict 300×1/2×0.5+300×1/2×0.7=180 events in the analysis for subgroup C, and so 420 + 180 = 600 events in the analysis for all patients. We then approximate the variance of the log hazard ratio for treatment resulting from Cox proportional hazard regressions, for the two subgroup analyses ($\sigma_{B}^{2}$ and $\sigma_{C}^{2}$) and the analysis for all patients ($\sigma_{A}^{2}$), as 4 divided by the predicted number of events in each analysis (Parmar et al. [29], their Equation 8).

We model the estimated log hazard ratios for the two subgroups B and C in the new trial as ${\hat{\mu }}_{B,\mathrm{new}}∼N\left(\mu_{B},\sigma_{B}^{2}\right)$ and ${\hat{\mu }}_{C,\mathrm{new}}∼N\left(\mu_{C},\sigma_{C}^{2}\right)$, where $\sigma_{B}^{2}$ and $\sigma_{C}^{2}$ are computed using the predicted number of events described in the preceding paragraph. We add ${\hat{\mu }}_{B,\mathrm{new}}$ and ${\hat{\mu }}_{C,\mathrm{new}}$ as quantities to be monitored when implementing the MCMC described in Section 3 and interpret them as predictions of the estimated log hazard ratios in the new trial that incorporate our prior beliefs and all uncertainty.

We also require a model for the estimated log hazard ratio ${\hat{\mu }}_{A,\mathrm{new}}$ in the entire new study population A=B∪C, for which we use ${\hat{\mu }}_{A,\mathrm{new}}=\pi {\hat{\mu }}_{B,\mathrm{new}}+\left(1-\pi \right){\hat{\mu }}_{C,\mathrm{new}}$. As before, π is the proportion of B patients. The quantity $\mu_{A}={\pi \mu }_{B}+\left(1-\pi \right)\mu_{C}$, from Equation (3) and discussed in Section 3, is therefore now applied to the corresponding estimates in the new study.

For trial design, we are usually interested in whether the new trial produces, in a frequentist sense, statistically significant results, both within subgroups and in its entire population. For this, we calculate test statistics for the new trial as $Z_{B,\mathrm{new}}={\hat{\mu }}_{B,\mathrm{new}}/\sigma_{B}$, $Z_{C,\mathrm{new}}={\hat{\mu }}_{C,\mathrm{new}}/\sigma_{C}$ and $Z_{A,\mathrm{new}}={\hat{\mu }}_{A,\mathrm{new}}/\sigma_{A}$. For example, if these test statistics are less than −1.96, then they are statistically significant, with the intended directional effect, at the 5% level when using a two‐sided hypothesis test. By exploring the implications of changing key design parameters for the trial's power (e.g. the overall sample size, proportion of patients recruited to each subgroup, hypothesis testing strategy) an examination of the joint predictive distribution of $\left(Z_{B,\mathrm{new}},Z_{C,\mathrm{new}},Z_{A,\mathrm{new}}\right)$ can inform key aspects of trial design.

**FIGURE 4:**
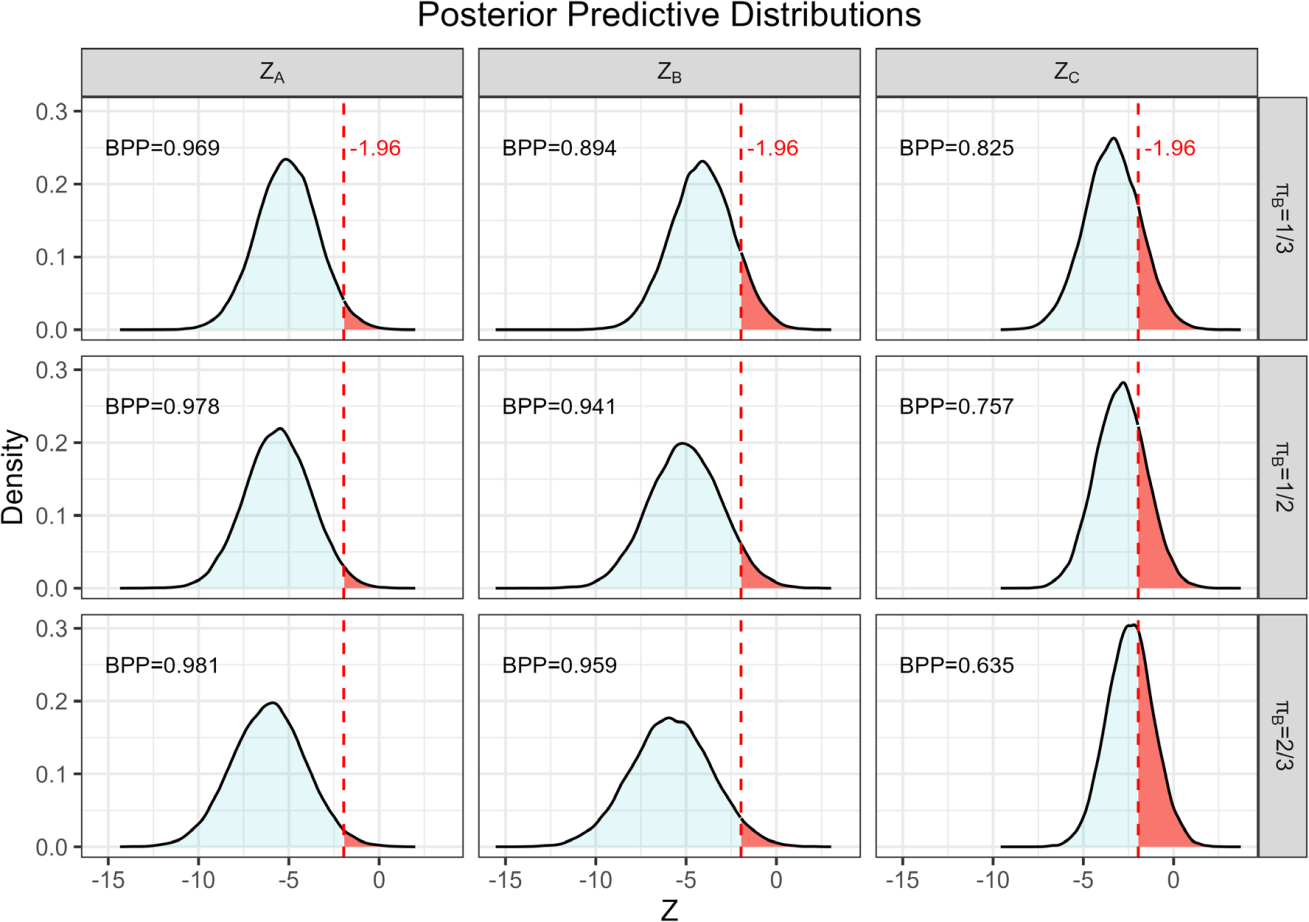
Posterior predictive distributions and Bayesian Predictive Powers (BPP) for the all‐comer population A (first column), population B (second column) and population C (third column) where 1/3 (first row), 1/2 (second row) and 2/3 (third row) of patients are sampled from population B.

In Figure 4, we show some illustrative results where the informative prior $\delta ∼N\left(-{0.122,0.334}^{2}\right)$ was applied to the Escudier et al. [27] data. Here, we show the posterior predictive distributions of $\left(Z_{B,\mathrm{new}},Z_{C,\mathrm{new}},Z_{A,\mathrm{new}}\right)$ and the probabilities that these are less than −1.96, that is, the posterior Bayesian Predictive Powers. This power in subgroup B (where the estimated treatment effect is largest; see Figure 3) increases as the proportion π of patients recruited from population B increases. Furthermore, this power for the A=B∪C population also increases slightly as π increases (although this is as high as around 97% even with π=1/3), also as expected because treatment efficacy is estimated to be greatest in the B population. However, increased power in the A and B populations achieved by increasing π from 1/3 to 1/2 is not improved much further by using π as large as 2/3, for which the power to detect an effect in the C population is as low as around 63%. These results indicate that, if achieving statistical significance in subgroup C is of importance, then it would be unwise to sample too small a proportion of patients from this population. This is because the large value of π=2/3 is very notably detrimental for achieving statistical significance in the C population whilst providing very little improvement in power in the other two populations of interest.

A complication is that different hierarchical hypothesis testing strategies are available to adjust for repeated testing in biomarker‐defined subgroups and the overall population [30, 31]. In order to be able to accommodate a particular sequential hypothesis testing strategy, we suggest examining the joint predictive distribution of $\left(Z_{B,\mathrm{new}},Z_{C,\mathrm{new}},Z_{A,\mathrm{new}}\right)$ more carefully. For example, if the hypothesis testing procedure is to test in subgroup B, only if there is a statistically significant result in A, we would then calculate the probability that $Z_{B,\mathrm{new}}$ is statistically significant conditional on this result. This probability is estimated from the proportion of times $Z_{B,\mathrm{new}}$ is less than the appropriate critical value in the sample of MCMC iterations for which $Z_{A,\mathrm{new}}$ is statistically significant.

## 6 Discussion

We have extended a recently published frequentist approach that explored biomarker subgroup analysis [1]. By incorporating prior distributions, and so adopting a Bayesian approach, the methodology proposed here is more versatile but it also makes more assumptions via the priors. Using prior information on the relationship between subgroups adds value to the assessment of subgroup differences, both in terms of design and analysis of such trials. This is because we can then perform statistical analyses, and so make decisions, that reflect our current understanding of the predictive value of biomarkers. In Section 4.1.1 we illustrated how to elicit an informative bivariate normal distribution for $\mu_{B}$ and $\mu_{C}$, and how to extend this to incorporate additional concerns of clinicians. These concerns include the possibility of no treatment effect in both populations and the potential dependence of the parameters in the prior distribution of $\mu_{B}$ on $\mu_{C}$. This novel, and unusual, joint prior distribution serves to illustrate that our Bayesian methods are able to incorporate both conventional and bespoke priors.

It is almost always of interest to compare Bayesian results to those from frequentist analyses, so that the impact of incorporating prior information can be better understood. Similarly comparing decisions for trial design, based on our methods to those from a frequentist viewpoint, will also be of interest, for similar reasons. Despite the advantages of Bayesian methods, use of priors remains controversial. Sensitivity analysis, either formal or informal that considers a range of possible prior specifications, will usually be desirable. An advantage of our approach is that it provides a framework, in which a wide variety of statistical analyses can be embedded, and so provides a conceptual basis for sensitivity analysis. If there is key prior information on a different scale, for example for the overall hazard ratio or relative hazard ratio between the two groups, then this can be transformed for inclusion in the statistical analysis within the MCMC. We have illustrated the use of informative prior distributions, motivated by similar trials, in our examples. Investigating the circumstances where markedly different conclusions are reached using different types of statistical analyses, either empirically or via simulation studies, would be a useful next step.

Our proposed approach may be especially useful when transitioning from one phase of clinical research to the next, so that key strategic decisions can be based on all available information. In particular, it may be helpful when designing trials at later phases, as illustrated using our second example in Section 5. These methods may be used with other overall treatment effects and alternatives to our approximation for $\mu_{A}$ may perform better in some instances. Our models can also be useful to quantify the probability of trial success, and so communicate the risks of particular decisions, to different stakeholders. Bayesian methods may also be valuable in situations where data are sparse, so that frequentist analysis that does not incorporate prior information will be subject to considerable uncertainty. However, issues relating to prior sensitivity become more pressing, because the priors may then dominate the analysis, and the importance of an assessment of this sensitivity increases.

There are clear differences between how regulatory agencies and reimbursers typically assess data. Regulators generally require sponsors to pre‐specify the analysis and to minimise the assumptions made. They ask for strong frequentist control of the Type 1 Error and the multiple testing generalisation thereof, the family‐wise error rate (FWER). This complicates the use of our proposals in Section 5 when designing a new trial. There is, however, greater variability in the standpoints of different reimbursers than among regulatory agencies. Reimbursers generally take into account costs, as well as benefits and risks. In many cases, even if a drug is approved in a wider population, it may only be deemed cost‐effective in a subgroup. Assuming that safety is not a concern, the posterior efficacies in populations B and C may justify the existing price of the drug, if it is already marketed for other indications, or motivate a price proposed for the first launch. In health technology assessment, more focus is typically placed on estimation and decision‐making, and Bayesian methodology is commonly used. The framework described in this article can therefore be of most direct use for reimbursers.

When statistical methods that allow the possibility of shrinking subgroup effects, to their mean, are applied in a regulatory setting, we suggest that it is reasonable to keep the overall perspective as close as possible to a standard regulatory pre‐specification, that uses a frequentist paradigm. Here we would first establish that the drug has efficacy, for example, showing statistical significance in the all‐comer or biomarker positive population. With placebo control, it is then usually biologically obvious, or at least very likely, that the null hypothesis for the biomarker negative population is incorrect; that is, that there is at least some efficacy of the drug in this population. Focus can then shift to estimation, and weighing benefits versus risks, for each subpopulation. Thus we also suggest that formal frequentist, or Bayesian, testing is not then a necessity in the biomarker negative population.

The EMA [8] Guideline on the investigation of subgroups in confirmatory clinical trials recommends that subgroup categories should be carefully considered at the planning stage and that factors defining subgroups of the target population may be put in three categories. The first category is where there is a strong reason to expect differences in treatment efficacy. The methodology in this paper is meant to be in spirit of this guidance. When using our framework, the sponsor identifies two disjoint subgroups that are expected to have unequal efficacy, to the extent that it is unclear whether the drug, even if it shows clear efficacy on average compared to control, should be approved in the full population or only in the biomarker positive subpopulation [32, 33]. In the situation where it is not feasible to generate enough data to make independent decisions for both subgroups, it has been common that the sponsor tests both the null hypothesis of no efficacy in all‐comers and the null hypothesis of no efficacy in the biomarker positive subpopulation. When these hypotheses are tested simultaneously (rather than hierarchically), the test proposed by Spiessens and Debois [34] can increase the power, while strongly controlling the FWER. A complication with this analysis, however, is that a statistically significant efficacy in the entire study population can be consistent with detrimental efficacy in the biomarker negative population. Regulators should therefore also assess the efficacy in the biomarker negative population. Our methodology explicitly facilitates this, using a pre‐specified prior that reflects biologic plausibility and previous data. As the EMA [8] states ‘Credibility depends on … a priori definition, the biologic plausibility … and replication’.

Our methods have used some statistical approximations. For example, we have used conventional normal approximations for distributions of the subgroup estimators, such as log hazard ratios, with estimation using MCMC. We have also proposed using a weighted average of the subgroup treatment effects to define an overall treatment effect that applies to the total patient population. This will be exact in some situations, for example, where the treatment effects are unadjusted mean differences. However, in our applications, we have modelled log hazard ratios, where the population level treatment is not simply a weighted average of subgroup treatment effects. This is because the hazard ratio is not collapsible. We are, however, content to use the proposed weighted average as an approximation in our modelling. Future work may explore how often this type of approximation is used and when it is adequate. Our position is that this is a reasonable, and widely applicable, approximation that will be adequate for the majority of applications. However, further work is planned to investigate this issue in detail and some preliminary mathematical work to understand this has already been undertaken. In practice, it is difficult to propose a more accurate result that is so widely applicable and simple to implement. With independent normal priors for two of the three linearly dependent parameters ($\mu_{B}$, $\mu_{C}$ and δ), analytic solutions can be derived for the posterior. By using a variety of different prior specifications, the results can range from effectively pooling the B and C groups to essentially treating them as unrelated.

Generalising our methods to incorporate more than two subgroups is an obvious next step. If there are many disjoint subgroups, then a hierarchical structure that assumes exchangeability, as sometimes proposed in the context of multi‐centre trials or random‐effects meta‐analysis, may be a feasible approach depending on the clinical setting. However if there are several, but not many, subgroups then variance components in hierarchical models are hard to identify. Informative priors may therefore be usefully employed in such instances but satisfactorily generalising our methods, to incorporate a third biomarker subgroup for example, may be challenging. Generalising all our prior distributions to incorporate several overlapping subgroups may also be difficult.

To summarise, we have shown how Bayesian methods may be useful in the design and analysis of trials that include a binary biomarker. We have implemented several different forms of prior distributions, for which computing codes are available in the Supporting Information. Some of the priors we have considered are informative, and motivated by similar trials, illustrating how information from other data sources can be incorporated in practice. We hope that our work will encourage others to explore the use of Bayesian methods for biomarker stratified trials.

## Conflicts of Interest

Dan Jackson, Fanni Zhang and Carl‐Fredrik Burman are employed by AstraZeneca.

## Supporting information


**Data S1** Supporting Information.

## Data Availability

The data that supports the findings of this study are available in the Supporting Information of this article.

## Appendix A

We assume that there no additional baseline covariates are included in model (2), so that ${\mathrm{\gamma X}}_{i}=0$. Hence, this model becomes

$$\eta_{i}=\beta_{0}+\beta_{1}T_{i}+\beta_{2}S_{i}+\delta S_{i}T_{i}.$$

To establish (conditional, on the model parameters) independence between the two subgroup estimates of treatment effect, we re‐parameterise $\beta_{4}=\beta_{0}+\beta_{2}$, and define $\mu_{B}=\beta_{1}+\delta$, and $\mu_{C}=\beta_{1}$ as in Section 2, so that this linear predictor is equivalent to

$$\eta_{i}=\left(\beta_{0}+\mu_{C}T_{i}\right)\left(1-S_{i}\right)+\left(\beta_{4}+\mu_{B}T_{i}\right)S_{i}.$$

We can now see that subjects with $S_{i}=1$, and so those from subgroup B, contribute terms to the likelihood that include $\beta_{4}$ and $\mu_{B}$. Similarly subjects with $S_{i}=0$, and so those from subgroup C, contribute terms to the likelihood that include $\beta_{0}$ and $\mu_{C}$. Hence, the likelihood factorises, where different sets of patients contribute to the estimation of $\mu_{B}$ and $\mu_{C}$, so that ${\hat{\mu }}_{B}$ and ${\hat{\mu }}_{C}$ are conditionally independent.

This conditional independence of ${\hat{\mu }}_{B}$ and ${\hat{\mu }}_{C}$ can be established explicitly for the linear regression model $Y_{i}=\beta_{0}+\beta_{1}T_{i}+\beta_{2}S_{i}+\delta S_{i}T_{i}+\varepsilon_{i}$, where $\varepsilon_{i}∼N\left(0,\sigma^{2}\right)$ and the proportion of patients in each subgroup B, s, is the same across treatment groups. The Gram matrix is then

$$X^{T}X=\left[\begin{array}{cccc} n & t & \mathrm{ns} & \mathrm{ts}\\ t & t & \mathrm{ts} & \mathrm{ts}\\ \mathrm{ns} & \mathrm{ts} & \mathrm{ns} & \mathrm{ts}\\ \mathrm{ts} & \mathrm{ts} & \mathrm{ts} & \mathrm{ts} \end{array}\right]$$

where n is the total number of subjects and t is the total number of treated patients $\left(T_{i}=1\right)$. We now write $1/D=\left(n-t\right)\left(1-s\right)$, so that

$${\left(X^{T}X\right)}^{-1}=\left[\begin{array}{cccc} D & -D & -D & D\\ -D & \mathrm{Dn}/t & D & -\mathrm{Dn}/t\\ -D & D & D/s & -D/s\\ D & -\mathrm{Dn}/t & -D/s & \mathrm{nD}/\mathrm{ts} \end{array}\right]$$

This matrix inverse is most easily established by checking that $X^{T}X{\left(X^{T}X\right)}^{-1}=I$ and ${\left(X^{T}X\right)}^{-1}X^{T}X=I$, where I is the 4×4 identity matrix. Hence, , implying (conditional) independence under the normality assumptions made by the linear regression model.

## Appendix B

Let $\hat{\mu }$ and $\sum_{l}$ be the estimate (the vector on the left hand side) and the covariance matrix in model (1), respectively. The prior distributions for $\mu_{C}∼N\left(0,100\right)$ and $\delta ∼N\left(a,b^{2}\right)$ are assumed to be independent. This implies a joint prior distribution for $\mu_{B}=\mu_{C}+\delta$ and $\mu_{C}$ of

(6)
$$\left(\begin{array}{c} \mu_{B}\\ \mu_{C} \end{array}\right)∼N\left(\left(\begin{array}{c} a\\ 0 \end{array}\right),\left(\begin{array}{cc} b^{2}+100, & 100\\ 100, & 100 \end{array}\right)\right).$$

Let $\mu_{p}$ and $\sum_{p}$ be the prior mean and covariance matrix in (6), respectively. Then, the joint posterior distribution of $\left(\mu_{B},\mu_{C}\right)$ is

(7)
$$\left(\begin{array}{c} \mu_{B}\\ \mu_{C} \end{array}\right)∼N\left({\left(\sum_{p}^{-1}+\sum_{l}^{-1}\right)}^{-1}\left(\sum_{p}^{-1}\mu_{p}+\sum_{l}^{-1}\hat{\mu }\right),{\left(\sum_{p}^{-1}+\sum_{l}^{-1}\right)}^{-1}\right).$$

The posterior distributions of $\mu_{B}$ and $\mu_{C}$ are then given as the marginal distributions from Equation (7). The posterior distribution of δ is simply the implied normal distribution of the linear combination $\mu_{B}-\mu_{C}$ from Equation (7).

## Appendix C

The probability density function of a mixture model is

(8)
$$\mathrm{pf}\left(\delta |M_{1}\right)+\left(1-p\right)f\left(\delta |M_{2}\right),$$

where p is the probability of model one ($M_{1}$) and $f\left(\delta |M_{1}\right)$ and $f\left(\delta |M_{2}\right)$ are the probability density functions of δ under model 1, and model 2 ($M_{2}$), respectively. In this appendix, we derive the posterior distributions of p and δ under the spike and slab prior, that is a special case of model (8).

For our spike and slab prior, we assume the prior specification $p∼U\left(0,1\right)$, $f\left(\delta |M_{1}\right)=\tau_{1}^{-1}\phi \left(\left(\delta -\delta_{1}\right)/\tau_{1}\right)$ and $f\left(\delta |M_{2}\right)=\tau_{2}^{-1}\phi \left(\left(\delta -\delta_{2}\right)/\tau_{2}\right)$, where $U\left(0,1\right)$ denotes the continuous uniform distribution from zero to one, and $\phi \left(.\right)$ is the standard normal probability density function. In Section 2.1.3, we assumed that $\delta_{1}=\delta_{2}=0$, and used particular values for $\tau_{1}$ and $\tau_{2}$, but we will derive results more generally where these four parameters may take other values. The joint prior density of p and δ is therefore

$$f\left(p,\delta \right)=\frac{p}{\sqrt{2\pi }\tau_{1}}\exp\left(-\frac{{\left(\delta -\delta_{1}\right)}^{2}}{2\tau_{1}^{2}}\right)+\frac{1-p}{\sqrt{2\pi }\tau_{2}}\exp\left(-\frac{{\left(\delta -\delta_{2}\right)}^{2}}{2\tau_{2}^{2}}\right)$$

for 0<p<1 and δ∈ℝ. We assume that we observe the estimate $\hat{\delta }∼N\left(\delta ,\sigma^{2}\right)$, so that the joint posterior distribution of p and δ is

(9)
$$\begin{array}{c} f\left(p,\delta |\hat{\delta }\right)\propto \frac{p}{2{\pi \sigma \tau }_{1}}\exp\left(-\frac{{\left(\hat{\delta }-\delta \right)}^{2}}{2\sigma^{2}}-\frac{{\left(\delta -\delta_{1}\right)}^{2}}{2\tau_{1}^{2}}\right)\\ \;+\frac{1-p}{2{\pi \sigma \tau }_{2}}\exp\left(-\frac{{\left(\hat{\delta }-\delta \right)}^{2}}{2\sigma^{2}}-\frac{{\left(\delta -\delta_{2}\right)}^{2}}{2\tau_{2}^{2}}\right) \end{array}$$

We now expand the terms in the exponents, complete the square and simplify, to express the posterior distribution in Equation (9) as

(10)
$$f\left(p,\delta |\hat{\delta }\right)\propto {\mathrm{pc}}_{1}\phi \left(\delta ;\frac{\tau_{1}^{2}\hat{\delta }+\sigma^{2}\delta_{1}}{\tau_{1}^{2}+\sigma^{2}},\frac{\sigma^{2}\tau_{1}^{2}}{\tau_{1}^{2}+\sigma^{2}}\right)+\left(1-p\right)c_{2}\phi \left(\delta ;\frac{\tau_{2}^{2}\hat{\delta }+\sigma^{2}\delta_{2}}{\tau_{2}^{2}+\sigma^{2}},\frac{\sigma^{2}\tau_{2}^{2}}{\tau_{2}^{2}+\sigma^{2}}\right)$$

where $\phi \left(.;\mu ,v^{2}\right)$ is the probability density function of a normal distribution with mean μ and variance $v^{2}$ and $c_{i}=\phi \left(\hat{\delta };\delta_{i},\sigma^{2}+\tau_{i}^{2}\right)$. Note that the normal densities in Equation (10) are the usual posteriors where the prior distributions are of the form $\delta ∼N\left(\delta_{i},\tau_{i}^{2}\right)$ and the observed data is $\hat{\delta }∼N\left(\delta ,\sigma^{2}\right)$.

Integrating out δ from Equarion (10) gives the marginal posterior distribution of p

(11)
$$f\left(p|\hat{\delta }\right)\propto c_{1}p+\left(1-p\right)c_{2},$$

By integrating Equation (11) over the interval $\left(0,1\right)$, we can evaluate the constant of proportionality required for the posterior probability density function to integrate to unity as $2/\left(c_{1}+c_{2}\right)$. Hence, we can express the marginal posterior density of p in Equation (11) as

$$f\left(p|\hat{\delta }\right)=\frac{2\left(c_{1}p+\left(1-p\right)c_{2}\right)}{c_{1}+c_{2}}$$

Also using the constant of proportionality $2/\left(c_{1}+c_{2}\right)$, and integrating out p, from Equation (10) gives

(12)
$$f\left(\delta |\hat{\delta }\right)=\frac{c_{1}\phi \left(\delta ;\frac{\tau_{1}^{2}\hat{\delta }+\sigma^{2}\delta_{1}}{\tau_{1}^{2}+\sigma^{2}},\frac{\sigma^{2}\tau_{1}^{2}}{\tau_{1}^{2}+\sigma^{2}}\right)+c_{2}\phi \left(\delta ;\frac{\tau_{2}^{2}\hat{\delta }+\sigma^{2}\delta_{2}}{\tau_{2}^{2}+\sigma^{2}},\frac{\sigma^{2}\tau_{2}^{2}}{\tau_{2}^{2}+\sigma^{2}}\right)}{c_{1}+c_{2}}$$

Noting again that that the normal densities in Equation (12) are the posteriors where the prior distributions are simply the spike or the slab priors, we can interpret this expression as a weighted average of the posterior distributions of δ under models $M_{1}$ and $M_{2}$, where the weights are $c_{1}$ and $c_{2}$, respectively. The weights are functions of the data $\hat{\delta }$ and the means and variances of the spike and slab priors.

Let model 1 be the slab. We, therefore, have a weight of $\phi \left(\hat{\delta };\delta_{1},\sigma^{2}+\tau_{1}^{2}\right)$ allocated to the posterior of the slab. As $\tau_{1}\to \infty$ $c_{1}\to 0$, so that a very diffuse prior for the slab will inevitably result in an analysis that is tantamount to assuming the spike with probability one, and no interaction between treatment and subgroup. It is therefore necessary to use a plausible value for $\tau_{1}^{2}$. Note that we have treated the variance components, $\tau_{1}^{2}$ and $\tau_{2}^{2}$, as fixed and known. If prior distributions were instead specified then we would need to marginalise over them.

## References

[1] K. Edgar , D. Jackson , K. Rhodes , T. Duffy , C. F. Burman , and L. Sharples , “Frequentist Rules for Regulatory Approval of Subgroups in Phase III Trials: A Fresh Look at an Old Problem,” Statistical Methods in Medical Research 30, no. 7 (2021): 1725–1743. 34077288 10.1177/09622802211017574 PMC8411475

[2] D. Altman and P. Royston , “The Cost of Dichotomising Continuous Variables,” British Medical Journal 332, no. 7549 (2006): 1080. 16675816 10.1136/bmj.332.7549.1080 PMC1458573

[3] M. Wechsler , S. Szefler , V. Ortega , et al., “Step‐Up Therapy in Black Children and Adults With Poorly Controlled Asthma,” New England Journal of Medicine 381, no. 13 (2019): 1227–1239. 31553835 10.1056/NEJMoa1905560 PMC7026584

[4] P. Asbell , M. Maguire , M. Pistilli , et al., “N‐3 Fatty Acid Supplementation for the Treatment of Dry Eye Disease,” New England Journal of Medicine 378, no. 18 (2018): 1681–1690. 29652551 10.1056/NEJMoa1709691 PMC5952353

[5] N. James , J. deBono , M. Spears , et al., “Abiraterone for Prostate Cancer Not Previously Treated With Hormone Therapy,” New England Journal of Medicine 377, no. 4 (2017): 338–351. 28578639 10.1056/NEJMoa1702900 PMC5533216

[6] N. Ballarini , Y. Chiu , F. Konig , M. Posch , and T. Jaki , “A Critical Review of Graphics for Subgroup Analyses in Clinical Trials,” Pharmaceutical Statistics 19, no. 5 (2020): 541–560. 32216035 10.1002/pst.2012 PMC8647927

[7] International Council for Harmonisation , “General Principles for Planning and Design of Regional Clinical Trials E17,” 2017, https://database.ich.org/sites/default/files/E17EWG_Step4_2017_1116.pdf.

[8] European Medicines Agency , “Guideline on the Investigation of Subgroups in Confirmatory Clinical Trials,” 2019, https://www.ema.europa.eu/en/documents/scientific‐guideline/guideline‐investigation‐subgroups‐confirmatory‐clinical‐trials_en.pdf.

[9] Food and Drug Administration , “Guidance for the Use of Bayesian Statistics in Medical Device Clinical Trials,” 2010, https://www.fda.gov/media/71512/download.

[10] R. Tidwell , S. Peng , M. Chen , D. Liu , Y. Yuan , and J. Lee , “Bayesian Clinical Trials at the University of Texas MD Anderson Cancer Center: An Update,” Clinical Trials 16, no. 6 (2019): 645–656. 31450957 10.1177/1740774519871471 PMC6904523

[11] M. Lavine , “Sensitivity in Bayesian Statistics: The Prior and the Likelihood,” Journal of the Americal Statistical Association 86, no. 414 (1991): 396–399.

[12] B. Bornkamp , D. Ohlssen , B. Magusson , and H. Schmidli , “Model Averaging for Treatment Effect Estimation in Subgroups,” Pharmaceutical Statistics 16, no. 2 (2017): 133–142. 27935199 10.1002/pst.1796

[13] N. Best , R. Price , I. Pouliquen , and O. Keene , “Assessing Efficacy in Important Subgroups in Confirmatory Trials: An Example Using Bayesian Dynamic Borrowing,” Pharmaceutical Statistics 20, no. 3 (2021): 551–562. 33475231 10.1002/pst.2093 PMC8247867

[14] H. Jones , D. Ohlssen , B. Neuenschwander , A. Racine , and M. Branson , “Bayesian Models for Subgroup Analysis in Clinical Trials,” Clinical Trials 8 (2011): 129–143. 21282293 10.1177/1740774510396933

[15] B. Efron , “Frequentist Accuracy of Bayesian Estimates,” Journal of the Royal Statistical Society Series B 77 (2015): 617–646. 10.1111/rssb.12080 PMC4467036 26089740

[16] K. L. Grantham , J. Kasza , S. Heritier , J. B. Carlin , and A. B. Forbes , “Evaluating the Performance of Bayesian and Restricted Maximum Likelihood Estimation for Stepped Wedge Cluster Randomized Trials With a Small Number of Clusters,” BMC Medical Research Methodology 22 (2022): 112. 35418034 10.1186/s12874-022-01550-8 PMC9009029

[17] P. C. Lambert , A. Sutton , P. Burton , K. Abrams , and D. Jones , “How Vague Is Vague? A Simulation Study of the Impact of the Use of Vague Prior Distributions in MCMC Using WinBUGS,” Statistics in Medicine 24, no. 15 (2005): 2401–2428. 16015676 10.1002/sim.2112

[18] L. Bojke , M. Soares , K. Claxton , et al., “Developing a Reference Protocol for Structured Expert Elicitation in Health‐Care Decision‐Making: A Mixed‐Methods Study,” Health Technology Assessment 25, no. 37 (2021): 1–124. 10.3310/hta25370 PMC8215568 34105510

[19] H. Ishwaran and J. Rao , “Spike and Slab Variable Selection: Frequentist and Bayesian Strategies,” Annals of Statistics 33, no. 2 (2005): 730–773.

[20] D. Lunn , A. Thomas , N. Best , and D. Spiegelhalter , “WinBUGS ‐ a Bayesian Modelling Framework: Concepts, Structure, and Extensibility,” Statistics and Computing 10 (2000): 325–337.

[21] C. Ryan , M. Smith , J. Bono , et al., “Abiraterone in Metastatic Prostate Cancer Without Previous Chemotherapy,” New England Medical Journal 368, no. 2 (2013): 138–148. 10.1056/NEJMoa1209096 PMC3683570 23228172

[22] D. Lunn , C. Jackson , N. Best , A. Thomas , and D. Spiegelhalter , The BUGS Book. A Practical Introduction to Bayesian Analysis (New York, NY: Taylor and Francis, 2012).

[23] J. Ibrahim , M. Chen , Y. Gwon , and F. Chen , “The Power Prior: Theory and Applications,” Statistics in Medicine 34, no. 28 (2015): 3724–3749. 26346180 10.1002/sim.6728 PMC4626399

[24] H. Schmidli , S. Gsteiger , S. Roychoudhury , A. O'Hagen , D. Spiegelhalter , and B. Neuenschwander , “Robust Meta‐Analytic‐Predictive Priors in Clinical Trials With Historical Control Information,” Biometrics 70, no. 4 (2014): 1023–1032. 25355546 10.1111/biom.12242

[25] C. Burman , E. Hermansson , D. Bock , S. Franzen , and D. Svensson , “Digital Twins and Bayesian Dynamic Borrowing: Two Recent Approaches for Incorporating Historical Control Data,” Pharmaceutical Statistics 23, no. 5 (2024): 611–629. 38439136 10.1002/pst.2376

[26] B. Holzhauer , L. Hampson , J. Gosling , et al., “Eliciting Judgements About Dependent Quantities Ofinterest: The SHeffield ELicitation Framework Extensionand Copula Methods Illustrated Using an Asthma Case Study,” Phamaceutical Statistics 21, no. 5 (2022): 1005–1021. 10.1002/pst.2212 35373454

[27] B. Escudier , T. Powles , R. Motzer , et al., “Cabozantinib, a New Standard of Care for Patients With Advanced Renal Cell Carcinoma and Bone Metastases? Subgroup Analysis of the METEOR Trial,” Journal of Clinical Oncology 36, no. 8 (2018): 765–772. 29309249 10.1200/JCO.2017.74.7352 PMC6804840

[28] T. Choueiri , T. Powles , M. Burotto , et al., “Nivolumab Plus Cabozantinib Versus Sunitinib for Advanced Renal‐Cell Carcinoma,” New England Journal of Medicine 384, no. 9 (2021): 829–841. 33657295 10.1056/NEJMoa2026982 PMC8436591

[29] M. Parmar , V. Torri , and L. Stewart , “Extracting Summary Statistics to Perform Meta‐Analyses of the Published Literature for Survival Endpoints,” Statistics in Medicine 17, no. 24 (1998): 2815–2834. 9921604 10.1002/(sici)1097-0258(19981230)17:24<2815::aid-sim110>3.0.co;2-8

[30] S. Matsui and J. Crowley , “Biomarker‐Stratified Phase III Clinical Trials: Enhancement With a Subgroup‐Focused Sequential Design,” Clinical Cancer Research 24, no. 5 (2018): 994–1001. 28887317 10.1158/1078-0432.CCR-17-1552

[31] M. Alosh , F. Bretz , and M. Huque , “Advanced Multiplicity Adjustment Methods in Clinical Trials,” Statistics in Medicine 33, no. 4 (2014): 693–713. 24105821 10.1002/sim.5974

[32] B. Freidlin and E. Korn , “A Problematic Biomarker Trial Design,” JNCI Journal of the National Cancer Institute 114, no. 2 (2022): 187–190. 34289052 10.1093/jnci/djab144 PMC8826527

[33] L. Meier McShane , M. Rothmann , and T. Fleming , “Finding the (Biomarker‐Defined) Subgroup of Patients Who Benefit From a Novel Therapy: No Time for Hide and Seek,” Clinical Trials 20, no. 4 (2023): 341–350. 37095696 10.1177/17407745231169692 PMC10523858

[34] B. Spiessens and M. Debois , “Adjusted Significance Levels for Subgroup Analyses in Clinical Trials,” Contemporary Clinical Trials 31, no. 6 (2010): 647–656. 20832503 10.1016/j.cct.2010.08.011

