# Supplementary material for: Bayesian Solutions for Assessing Differential Effects in Biomarker Positive and Negative Subgroups
Source: Pharm Stat. 2024 Nov 25;24(2):e2456. doi: 10.1002/pst.2456 (PMC11893291; doi:10.1002/pst.2456)
Supplement: Supplementary file 1 — Data S1 Supporting Information. [file PST-24-0-s001.zip › pst-23-0103-File006.html]

Bayesian subgroup examples with all priors


Code 

- Show All Code
- Hide All Code

# Bayesian subgroup examples with all priors

#### 2024-08-20

This document demonstrates the application of various Bayesian prior distributions through two illustrative examples. Our methodology is explored in three main sections: initially, we examine three fundamental prior distributions for the parameter \(\delta\) to assess the differential treatment effects between two subgroups. Subsequently, we design a trial similar to the second example, incorporating an informative prior to guide the design process. The final section expands our methodology by introducing additional prior distributions and enhancing our analysis with visual representations of the results.

### 1. Fundamental prior distributions for \(\delta\)

In the initial phase of our exploration, we focus on three core prior distributions applied to \(\delta\). These distributions provide a foundation for understanding how prior beliefs can influence the evaluation of treatment effects.

- Discrete prior distribution for \(\delta\)
- Normal prior distribution for \(\delta\)
- Spike and slab prior distribution for \(\delta\)

#### 1.1 STAMPEDE

1917 patients enrolled, 915 with nonmetastatic disease (subgroup B, smaller treatment effect) and 1002 with metastatic disease (complement C, larger treatment effect). The hazard ratio (HR) was 0.75 (95% CI, 0.48-1.18) in patients with non-metastatic disease and 0.61 (95% CI, 0.49-0.75) in those with metastatic disease.

First, derive the standard errors for each subgroup based on the confidence intervals and sample sizes.

```
# create a summary table for HR and calculate SE
gen.table <- function(n.vec, HR.vec, HR.LCI.vec, HR.UCI.vec, group.char) {
  HR.CI <- paste0("(", HR.LCI.vec, ", ", HR.UCI.vec, ")")
  logHR <- log(HR.vec)
  SE.logHR <- (log(HR.UCI.vec) - log(HR.LCI.vec)) / (2 * 1.96)
  
  HR.table <- data.frame(
    Subgroup = group.char,
    NumPatients = n.vec,
    `HR (95% CI) `= paste0(HR.vec, " ", HR.CI),
    logHR = logHR,
    SE.logHR = SE.logHR,
    check.names = F
  )
  return(HR.table)
}

# HR info from example 1
n.vec <- c(915, 1002)
HR.vec <- c(0.75, 0.61)
HR.LCI.vec <- c(0.48, 0.49)
HR.UCI.vec <- c(1.18, 0.75)
group.char <- c("Non-Metastatic (B)", "Metastatic (C)")

# generate the HR summary
HR.table <- gen.table(n.vec, HR.vec, HR.LCI.vec, HR.UCI.vec, group.char)
HR.table.CI <- HR.table
HR.table.CI[, "logHR.LCI"] <- log(HR.LCI.vec)
HR.table.CI[, "logHR.UCI"] <- log(HR.UCI.vec)

estB <- HR.table$logHR[1]
estC <- HR.table$logHR[2]
sB <- HR.table$SE.logHR[1]
sC <- HR.table$SE.logHR[2]
est.ex1 <- data.frame(
  example = "STAMPEDE", 
  estB = estB, 
  estC = estC, 
  sB = sB, 
  sC = sC
)

knitr::kable(HR.table, format = "simple", digits = 2, caption = "STAMPEDE HR Summary, Metastatic vs Non-Metastatic")
```

STAMPEDE HR Summary, Metastatic vs Non-Metastatic

| Subgroup | NumPatients | HR (95% CI) | logHR | SE.logHR |
| --- | --- | --- | --- | --- |
| Non-Metastatic (B) | 915 | 0.75 (0.48, 1.18) | -0.29 | 0.23 |
| Metastatic (C) | 1002 | 0.61 (0.49, 0.75) | -0.49 | 0.11 |

##### 1.1.1 Discrete prior for \(\delta\)

```
est.ex1
```

```
##    example       estB       estC        sB        sC
## 1 STAMPEDE -0.2876821 -0.4942963 0.2294601 0.1085887
```

```
# discrete prior for delta 
out.disc <- fit.disc(
  estB = estB, estC = estC, 
  sB = sB, sC = sC, 
  disc.seq = seq(-2, 2, by = 0.1)
)
```

```
## module glm loaded
```

```
## Compiling model graph
##    Resolving undeclared variables
##    Allocating nodes
## Graph information:
##    Observed stochastic nodes: 2
##    Unobserved stochastic nodes: 2
##    Total graph size: 98
## 
## Initializing model
```

```
print(out.disc$sum)
```

```
## Inference for Bugs model at "C:/Users/kpvp028/AppData/Local/Temp/RtmpIBhp0H/model653028a2257c.txt", fit using jags,
##  2 chains, each with 50000 iterations (first 20000 discarded), n.thin = 2
##  n.sims = 30000 iterations saved
##          mu.vect sd.vect   2.5%    25%    50%    75%  97.5%  Rhat n.eff
## delta      0.208   0.254 -0.300  0.000  0.200  0.400  0.700 1.001 14000
## m         23.079   2.540 18.000 21.000 23.000 25.000 28.000 1.001 15000
## muB       -0.286   0.230 -0.735 -0.440 -0.286 -0.131  0.163 1.001  9400
## muC       -0.494   0.109 -0.706 -0.567 -0.495 -0.421 -0.279 1.001 30000
## deviance  -1.708   2.015 -3.669 -3.099 -2.294 -0.991  3.879 1.001 30000
## 
## For each parameter, n.eff is a crude measure of effective sample size,
## and Rhat is the potential scale reduction factor (at convergence, Rhat=1).
## 
## DIC info (using the rule, pD = var(deviance)/2)
## pD = 2.0 and DIC = 0.3
## DIC is an estimate of expected predictive error (lower deviance is better).
```

```
densityplot(out.disc$results[, "delta"])
```

```
hist(out.disc$results[, "m"])
```

```
densityplot(out.disc$results[, "muB"])
```

```
densityplot(out.disc$results[, "muC"])
```

##### 1.1.2 Normal prior for \(\delta\)

```
# normal prior for delta
out.cont <- fit.cont(
  estB = estB, estC = estC, 
  sB = sB, sC = sC, 
  prior.mean = 0, prior.se = 10
)
```

```
## Compiling model graph
##    Resolving undeclared variables
##    Allocating nodes
## Graph information:
##    Observed stochastic nodes: 2
##    Unobserved stochastic nodes: 2
##    Total graph size: 20
## 
## Initializing model
```

```
out.cont$sum
```

```
## Inference for Bugs model at "C:/Users/kpvp028/AppData/Local/Temp/RtmpIBhp0H/model6530441c6ca7.txt", fit using jags,
##  2 chains, each with 50000 iterations (first 20000 discarded), n.thin = 2
##  n.sims = 30000 iterations saved
##          mu.vect sd.vect   2.5%    25%    50%    75%  97.5%  Rhat n.eff
## delta      0.209   0.254 -0.287  0.038  0.210  0.381  0.707 1.001  4900
## muB       -0.285   0.229 -0.734 -0.440 -0.285 -0.130  0.168 1.001  8900
## muC       -0.494   0.109 -0.706 -0.567 -0.493 -0.422 -0.281 1.001  8200
## deviance  -1.707   2.006 -3.658 -3.134 -2.336 -0.943  3.704 1.001 30000
## 
## For each parameter, n.eff is a crude measure of effective sample size,
## and Rhat is the potential scale reduction factor (at convergence, Rhat=1).
## 
## DIC info (using the rule, pD = var(deviance)/2)
## pD = 2.0 and DIC = 0.3
## DIC is an estimate of expected predictive error (lower deviance is better).
```

```
densityplot(out.cont$results[, "delta"])
```

```
densityplot(out.cont$results[, "muB"])
```

```
densityplot(out.cont$results[, "muC"])
```

##### 1.1.3 Spike and slab prior for \(\delta\)

```
# spike and slab prior for delta
out.ss <- fit.ss(
  estB = estB, estC = estC, 
  sB = sB, sC = sC
)
```

```
## Compiling model graph
##    Resolving undeclared variables
##    Allocating nodes
## Graph information:
##    Observed stochastic nodes: 2
##    Unobserved stochastic nodes: 5
##    Total graph size: 23
## 
## Initializing model
```

```
out.ss$sum
```

```
## Inference for Bugs model at "C:/Users/kpvp028/AppData/Local/Temp/RtmpIBhp0H/model653038ef531c.txt", fit using jags,
##  2 chains, each with 50000 iterations (first 20000 discarded), n.thin = 2
##  n.sims = 30000 iterations saved
##          mu.vect sd.vect    2.5%    25%    50%    75%  97.5%  Rhat n.eff
## Islab      0.038   0.192   0.000  0.000  0.000  0.000  1.000 1.001 30000
## d[1]       0.000   0.010  -0.019 -0.006  0.000  0.007  0.020 1.001 30000
## d[2]       0.051   9.804 -19.404 -6.428  0.136  6.487 19.640 1.001 30000
## delta      0.008   0.065  -0.021 -0.006  0.001  0.008  0.105 1.001 30000
## muB       -0.451   0.112  -0.654 -0.522 -0.455 -0.386 -0.227 1.001 30000
## muC       -0.459   0.099  -0.654 -0.525 -0.459 -0.392 -0.264 1.001 30000
## pick       1.038   0.192   1.000  1.000  1.000  1.000  2.000 1.001 30000
## prob       0.348   0.245   0.014  0.141  0.307  0.522  0.875 1.001 15000
## deviance  -2.028   1.452  -3.117 -2.934 -2.580 -1.692  2.086 1.001 30000
## 
## For each parameter, n.eff is a crude measure of effective sample size,
## and Rhat is the potential scale reduction factor (at convergence, Rhat=1).
## 
## DIC info (using the rule, pD = var(deviance)/2)
## pD = 1.1 and DIC = -1.0
## DIC is an estimate of expected predictive error (lower deviance is better).
```

```
densityplot(out.ss$results[, "delta"])
```

```
densityplot(out.ss$results[, "d[1]"])  # spike at zero
```

```
densityplot(out.ss$results[, "d[2]"])  # slab
```

```
densityplot(out.ss$results[, "muB"])
```

```
densityplot(out.ss$results[, "muC"])
```

```
densityplot(out.ss$results[, "pick"])
```

```
densityplot(out.ss$results[, "prob"])
```

##### 1.1.4 Normal prior for \(\delta\) and informative prior for \(\mu\_C\)

```
# normal prior for delta + informative muC prior
# use CI from Ryan et al to provide informative prior for muC
# This is HR=0.75
# 95% CI 0.61 to 0.93
# therefore mean is log(0.75) = -0.2876821
# se = (log(0.93)-log(0.61))/(2*1.96) =  0.1075831
HR.info <- 0.75
HR.UCI.info <- 0.93
HR.LCI.info <- 0.61
muC.prior.mean.info <- log(HR.info)
muC.prior.se.info <- (log(HR.UCI.info) - log(HR.LCI.info)) / (2 * 1.96)
print(paste0("muC.prior.mean.info: ", round(muC.prior.mean.info, 3)))
```

```
## [1] "muC.prior.mean.info: -0.288"
```

```
print(paste0("muC.prior.se.info: ", round(muC.prior.se.info, 3)))
```

```
## [1] "muC.prior.se.info: 0.108"
```

```
out.cont.info <- fit.cont(
  estB = estB, estC = estC, 
  sB = sB, sC = sC, 
  prior.mean = 0, prior.se = 10, 
  muC.prior.mean = muC.prior.mean.info,
  muC.prior.se = muC.prior.se.info
)
```

```
## Compiling model graph
##    Resolving undeclared variables
##    Allocating nodes
## Graph information:
##    Observed stochastic nodes: 2
##    Unobserved stochastic nodes: 2
##    Total graph size: 22
## 
## Initializing model
```

```
out.cont.info$sum
```

```
## Inference for Bugs model at "C:/Users/kpvp028/AppData/Local/Temp/RtmpIBhp0H/model65305f3d6c17.txt", fit using jags,
##  2 chains, each with 50000 iterations (first 20000 discarded), n.thin = 2
##  n.sims = 30000 iterations saved
##          mu.vect sd.vect   2.5%    25%    50%    75%  97.5%  Rhat n.eff
## delta      0.101   0.240 -0.374 -0.059  0.100  0.262  0.571 1.001  9500
## muB       -0.289   0.228 -0.735 -0.442 -0.288 -0.136  0.159 1.001  6300
## muC       -0.390   0.077 -0.541 -0.442 -0.390 -0.338 -0.240 1.001 30000
## deviance  -1.297   2.079 -3.623 -2.843 -1.834 -0.330  4.031 1.001 30000
## 
## For each parameter, n.eff is a crude measure of effective sample size,
## and Rhat is the potential scale reduction factor (at convergence, Rhat=1).
## 
## DIC info (using the rule, pD = var(deviance)/2)
## pD = 2.2 and DIC = 0.9
## DIC is an estimate of expected predictive error (lower deviance is better).
```

```
densityplot(out.cont.info$results[, "delta"])
```

```
densityplot(out.cont.info$results[, "muB"])
```

```
densityplot(out.cont.info$results[, "muC"])
```

Summarize the mean and 95% CI for the posterior treatment effect in the below table.

```
vars <- c("muB", "muC", "delta")
methods <- c("Frequentist", "Normal (vague)", "Discrete", "Spike/Slab", "Normal (info)")
out.list <- vector("list", length(methods) - 1)
out.list[[1]] <- out.cont
out.list[[2]] <- out.disc
out.list[[3]] <- out.ss
out.list[[4]] <- out.cont.info
names(out.list) <- methods[-1]

HR.table.CI1 <- HR.table.CI
out.list1 <- out.list

gen.sum <- function(out.list, ndigit = 3, params = vars, HR.table.CI=NULL) {
  cnames <- c("Method/Prior", "Parameter", "Mean", "SD", "Median", "95%LCI", "95%UCI")
  
  # initialize an empty list for summary data frames
  sum.list <- list()
  
  # process summaries for each method in out.list
  for (i in 1:length(out.list)) {
    out <- out.list[[i]]
    method.sum <- data.frame(
      "Method/Prior" = rep(names(out.list)[i], length(params)),
      "Parameter" = params,
      "Mean" = NA,
      "SD" = NA,
      "Median" = NA,
      "95%LCI" = NA,
      "95%UCI" = NA,
      check.names = F
    )
    
    for (param in params) {
      param.sum <- out$sum$BUGSoutput$summary[param, ]
      method.sum[method.sum$Parameter == param, c("Mean", "SD", "Median", "95%LCI", "95%UCI")] <- 
        param.sum[c("mean", "sd", "50%", "2.5%", "97.5%")]
    }
    
    sum.list[[i]] <- method.sum
  }
  # combine all summaries into a single data frame
  res <- do.call(rbind, sum.list)
  
  if(!is.null(HR.table.CI)){
    # frequentist summary
    mean.diff <- HR.table.CI$logHR[1] - HR.table.CI$logHR[2]
    se.diff <- sqrt(HR.table.CI$SE.logHR[1]^2 + HR.table.CI$SE.logHR[2]^2)
    freq.sum <- data.frame(
      "Method/Prior" = rep("Frequentist", length(params)),
      "Parameter" = params,
      "Mean" = c(HR.table.CI$logHR[1], HR.table.CI$logHR[2], mean.diff),
      "SD" = c(HR.table.CI$SE.logHR[1], HR.table.CI$SE.logHR[2], se.diff),
      "Median" = NA,
      "95%LCI" = c(HR.table.CI$logHR.LCI[1], HR.table.CI$logHR.LCI[2], mean.diff - 1.96 * se.diff),
      "95%UCI" = c(HR.table.CI$logHR.UCI[1], HR.table.CI$logHR.UCI[2], mean.diff + 1.96 * se.diff),
      check.names = F
    )
    # add freq.sum into results
    res <- rbind(freq.sum, res)
  }

  # format numeric columns
  cols.numeric <- c("Mean", "SD", "Median", "95%LCI", "95%UCI")
  fmt <- paste0("%.", ndigit, "f")
  res[cols.numeric] <- sapply(res[cols.numeric], function(x) sprintf(fmt, as.numeric(x)))
  
  # add 95% CI column
  res$`95%CI` <- paste0("(", res$`95%LCI`, ", ", res$`95%UCI`, ")")
  
  # select and rearrange columns
  res.fmt <- res[, c("Method/Prior", "Parameter", "Mean", "SD", "Median", "95%CI")]
  
  # return list of original and formatted summaries
  out <- list(res = res, res.fmt = res.fmt)
  names(out) <- c("res", "res.fmt")
  return(out)
}

# generate the summary table
out.ex1 <- gen.sum(out.list = out.list1, params = vars, HR.table.CI = HR.table.CI1)
res.ex1 <- out.ex1$res
res.fmt.ex1 <- out.ex1$res.fmt
res.fmt.ex1
```

```
##      Method/Prior Parameter   Mean    SD Median            95%CI
## 1     Frequentist       muB -0.288 0.229     NA  (-0.734, 0.166)
## 2     Frequentist       muC -0.494 0.109     NA (-0.713, -0.288)
## 3     Frequentist     delta  0.207 0.254     NA  (-0.291, 0.704)
## 4  Normal (vague)       muB -0.285 0.229 -0.285  (-0.734, 0.168)
## 5  Normal (vague)       muC -0.494 0.109 -0.493 (-0.706, -0.281)
## 6  Normal (vague)     delta  0.209 0.254  0.210  (-0.287, 0.707)
## 7        Discrete       muB -0.286 0.230 -0.286  (-0.735, 0.163)
## 8        Discrete       muC -0.494 0.109 -0.495 (-0.706, -0.279)
## 9        Discrete     delta  0.208 0.254  0.200  (-0.300, 0.700)
## 10     Spike/Slab       muB -0.451 0.112 -0.455 (-0.654, -0.227)
## 11     Spike/Slab       muC -0.459 0.099 -0.459 (-0.654, -0.264)
## 12     Spike/Slab     delta  0.008 0.065  0.001  (-0.021, 0.105)
## 13  Normal (info)       muB -0.289 0.228 -0.288  (-0.735, 0.159)
## 14  Normal (info)       muC -0.390 0.077 -0.390 (-0.541, -0.240)
## 15  Normal (info)     delta  0.101 0.240  0.100  (-0.374, 0.571)
```

#### 1.2 METEOR

Of the 658 patients enrolled, 142 belonged to a pre-specified subgroup having bone metastasis. We label this subgroup B and the complement C, although the dividing factor is strictly speaking not a biomarker but another baseline covariate. Esculier *et al* (2018) provides estimates and confidence intervals for the OS hazard ratio (HR) comparing the two treatments in the different sub-populations.

```
# OS HR info from example 2
n.vec <- c(142,516)
HR.vec <- c(0.54,0.71)
HR.LCI.vec <- c(0.34,0.55)
HR.UCI.vec <- c(0.84,0.91)
group.char <- c("With Bone Metastatic (B)", "Without Bone Metastatic (C)")

# generate the HR summary for OS
HR.table <- gen.table(n.vec, HR.vec, HR.LCI.vec, HR.UCI.vec, group.char)
HR.table.CI <- HR.table
HR.table.CI[,"logHR.LCI"] <- log(HR.LCI.vec)
HR.table.CI[,"logHR.UCI"] <- log(HR.UCI.vec)

estB <- HR.table$logHR[1]
estC <- HR.table$logHR[2]
sB <- HR.table$SE.logHR[1]
sC <- HR.table$SE.logHR[2]
est.ex2 <- data.frame(
  example = "METEOR", 
  estB = estB, 
  estC = estC, 
  sB = sB, 
  sC = sC
)

knitr::kable(HR.table, "simple", digits = 2, caption = "METEOR HR Summary, With/Without Bone Metastatic")
```

METEOR HR Summary, With/Without Bone Metastatic

| Subgroup | NumPatients | HR (95% CI) | logHR | SE.logHR |
| --- | --- | --- | --- | --- |
| With Bone Metastatic (B) | 142 | 0.54 (0.34, 0.84) | -0.62 | 0.23 |
| Without Bone Metastatic (C) | 516 | 0.71 (0.55, 0.91) | -0.34 | 0.13 |

##### 1.2.1 Discrete prior for \(\delta\)

```
est.ex2
```

```
##   example       estB       estC        sB        sC
## 1  METEOR -0.6161861 -0.3424903 0.2307286 0.1284506
```

```
# discrete prior for delta 
out.disc <- fit.disc(
  estB = estB, estC = estC, 
  sB = sB, sC = sC, 
  disc.seq = seq(-2, 2, by = 0.1)
)
```

```
## Compiling model graph
##    Resolving undeclared variables
##    Allocating nodes
## Graph information:
##    Observed stochastic nodes: 2
##    Unobserved stochastic nodes: 2
##    Total graph size: 98
## 
## Initializing model
```

```
print(out.disc$sum)
```

```
## Inference for Bugs model at "C:/Users/kpvp028/AppData/Local/Temp/RtmpIBhp0H/model6530580151c7.txt", fit using jags,
##  2 chains, each with 50000 iterations (first 20000 discarded), n.thin = 2
##  n.sims = 30000 iterations saved
##          mu.vect sd.vect   2.5%    25%    50%    75%  97.5%  Rhat n.eff
## delta     -0.274   0.264 -0.800 -0.500 -0.300 -0.100  0.200 1.001 30000
## m         18.257   2.644 13.000 16.000 18.000 20.000 23.000 1.001 30000
## muB       -0.617   0.231 -1.071 -0.773 -0.615 -0.462 -0.169 1.001 30000
## muC       -0.343   0.128 -0.593 -0.429 -0.343 -0.256 -0.092 1.001 30000
## deviance  -1.366   1.994 -3.309 -2.800 -1.970 -0.610  4.091 1.001 30000
## 
## For each parameter, n.eff is a crude measure of effective sample size,
## and Rhat is the potential scale reduction factor (at convergence, Rhat=1).
## 
## DIC info (using the rule, pD = var(deviance)/2)
## pD = 2.0 and DIC = 0.6
## DIC is an estimate of expected predictive error (lower deviance is better).
```

```
densityplot(out.disc$results[, "delta"])
```

```
hist(out.disc$results[, "m"])
```

```
densityplot(out.disc$results[, "muB"])
```

```
densityplot(out.disc$results[, "muC"])
```

##### 1.2.2 Normal prior for \(\delta\)

```
# normal prior for delta
out.cont <- fit.cont(
  estB = estB, estC = estC, 
  sB = sB, sC = sC, 
  prior.mean = 0, prior.se = 10
)
```

```
## Compiling model graph
##    Resolving undeclared variables
##    Allocating nodes
## Graph information:
##    Observed stochastic nodes: 2
##    Unobserved stochastic nodes: 2
##    Total graph size: 20
## 
## Initializing model
```

```
out.cont$sum
```

```
## Inference for Bugs model at "C:/Users/kpvp028/AppData/Local/Temp/RtmpIBhp0H/model6530f75fb2.txt", fit using jags,
##  2 chains, each with 50000 iterations (first 20000 discarded), n.thin = 2
##  n.sims = 30000 iterations saved
##          mu.vect sd.vect   2.5%    25%    50%    75%  97.5%  Rhat n.eff
## delta     -0.274   0.265 -0.792 -0.452 -0.273 -0.096  0.245 1.001 30000
## muB       -0.616   0.232 -1.073 -0.772 -0.615 -0.462 -0.160 1.001 30000
## muC       -0.342   0.128 -0.595 -0.428 -0.343 -0.256 -0.091 1.001 23000
## deviance  -1.351   2.023 -3.309 -2.792 -1.978 -0.568  4.051 1.001 30000
## 
## For each parameter, n.eff is a crude measure of effective sample size,
## and Rhat is the potential scale reduction factor (at convergence, Rhat=1).
## 
## DIC info (using the rule, pD = var(deviance)/2)
## pD = 2.0 and DIC = 0.7
## DIC is an estimate of expected predictive error (lower deviance is better).
```

```
densityplot(out.cont$results[, "delta"])
```

```
densityplot(out.cont$results[, "muB"])
```

```
densityplot(out.cont$results[, "muC"])
```

##### 1.2.3 Spike and slab prior for \(\delta\)

```
# spike and slab prior for delta
out.ss <- fit.ss(
  estB = estB, estC = estC, 
  sB = sB, sC = sC
)
```

```
## Compiling model graph
##    Resolving undeclared variables
##    Allocating nodes
## Graph information:
##    Observed stochastic nodes: 2
##    Unobserved stochastic nodes: 5
##    Total graph size: 23
## 
## Initializing model
```

```
out.ss$sum
```

```
## Inference for Bugs model at "C:/Users/kpvp028/AppData/Local/Temp/RtmpIBhp0H/model653053aa6776.txt", fit using jags,
##  2 chains, each with 50000 iterations (first 20000 discarded), n.thin = 2
##  n.sims = 30000 iterations saved
##          mu.vect sd.vect    2.5%    25%    50%    75%  97.5%  Rhat n.eff
## Islab      0.041   0.199   0.000  0.000  0.000  0.000  1.000 1.001 30000
## d[1]       0.000   0.010  -0.020 -0.007  0.000  0.006  0.019 1.001 30000
## d[2]      -0.155   9.795 -19.666 -6.560 -0.206  6.272 19.184 1.001 30000
## delta     -0.012   0.078  -0.207 -0.008 -0.001  0.006  0.020 1.002 14000
## muB       -0.416   0.127  -0.674 -0.491 -0.412 -0.333 -0.183 1.001 30000
## muC       -0.404   0.114  -0.626 -0.481 -0.405 -0.328 -0.179 1.001 30000
## pick       1.041   0.199   1.000  1.000  1.000  1.000  2.000 1.001 30000
## prob       0.344   0.244   0.011  0.137  0.302  0.516  0.871 1.001 29000
## deviance  -1.291   1.435  -2.399 -2.175 -1.827 -0.951  2.808 1.001 30000
## 
## For each parameter, n.eff is a crude measure of effective sample size,
## and Rhat is the potential scale reduction factor (at convergence, Rhat=1).
## 
## DIC info (using the rule, pD = var(deviance)/2)
## pD = 1.0 and DIC = -0.3
## DIC is an estimate of expected predictive error (lower deviance is better).
```

```
densityplot(out.ss$results[, "delta"])
```

```
densityplot(out.ss$results[, "d[1]"])  # spike at zero
```

```
densityplot(out.ss$results[, "d[2]"])  # slab
```

```
densityplot(out.ss$results[, "muB"])
```

```
densityplot(out.ss$results[, "muC"])
```

```
densityplot(out.ss$results[, "pick"])
```

```
densityplot(out.ss$results[, "prob"])
```

##### 1.2.4 Normal informative prior for \(\delta\)

```
# nomral prior for delta + informative delta prior
# use CI from Choueiri et al to provide informative prior for delta
# This is HR.B=0.54, HR.C=0.61
# HR.B.CI=c(0.32,0.92), HR.C.CI=(0.41,0.89)
# therefore mean for delta is log(HR.B)-log(HR.C) = -0.122
# se = (log(0.93)-log(0.61))/(2*1.96) =  0.334
HRB.info <- 0.54
HRC.info <- 0.61
HRB.UCI.info <- 0.92
HRB.LCI.info <- 0.32
HRC.UCI.info <- 0.89
HRC.LCI.info <- 0.41
delta.prior.mean.info <- log(HRB.info) - log(HRC.info)
delta.prior.se.info <- sqrt(((log(HRB.UCI.info) - log(HRB.LCI.info)) / (2 * 1.96))^2 + 
                            ((log(HRC.UCI.info) - log(HRC.LCI.info)) / (2 * 1.96))^2)
print(paste0("delta.prior.mean.info: ", round(delta.prior.mean.info, 3)))
```

```
## [1] "delta.prior.mean.info: -0.122"
```

```
print(paste0("delta.prior.se.info: ", round(delta.prior.se.info, 3)))
```

```
## [1] "delta.prior.se.info: 0.334"
```

```
out.cont.info<-fit.cont(
  estB = estB, estC = estC, 
  sB = sB, sC = sC, 
  prior.mean = delta.prior.mean.info,
  prior.se = delta.prior.se.info,
  muC.prior.mean = 0, muC.prior.se = 10
)
```

```
## Compiling model graph
##    Resolving undeclared variables
##    Allocating nodes
## Graph information:
##    Observed stochastic nodes: 2
##    Unobserved stochastic nodes: 2
##    Total graph size: 22
## 
## Initializing model
```

```
out.cont.info$sum
```

```
## Inference for Bugs model at "C:/Users/kpvp028/AppData/Local/Temp/RtmpIBhp0H/model65306d556dc5.txt", fit using jags,
##  2 chains, each with 50000 iterations (first 20000 discarded), n.thin = 2
##  n.sims = 30000 iterations saved
##          mu.vect sd.vect   2.5%    25%    50%    75%  97.5%  Rhat n.eff
## delta     -0.216   0.207 -0.619 -0.356 -0.215 -0.077  0.190 1.001 30000
## muB       -0.571   0.195 -0.951 -0.703 -0.571 -0.439 -0.191 1.001 30000
## muC       -0.355   0.123 -0.598 -0.438 -0.354 -0.272 -0.117 1.001 30000
## deviance  -1.689   1.710 -3.319 -2.887 -2.219 -1.071  2.899 1.001 30000
## 
## For each parameter, n.eff is a crude measure of effective sample size,
## and Rhat is the potential scale reduction factor (at convergence, Rhat=1).
## 
## DIC info (using the rule, pD = var(deviance)/2)
## pD = 1.5 and DIC = -0.2
## DIC is an estimate of expected predictive error (lower deviance is better).
```

```
densityplot(out.cont.info$results[, "delta"])
```

```
densityplot(out.cont.info$results[, "muB"])
```

```
densityplot(out.cont.info$results[, "muC"])
```

Summarize the mean and 95% CI for the posterior treatment effect in the below table.

```
out.list <- vector("list", length(methods) - 1)
out.list[[1]] <- out.cont
out.list[[2]] <- out.disc
out.list[[3]] <- out.ss
out.list[[4]] <- out.cont.info
names(out.list) <- methods[-1]

HR.table.CI2 <- HR.table.CI
out.list2 <- out.list
out.ex2 <- gen.sum(out.list = out.list2, HR.table.CI = HR.table.CI2, params = vars)
res.ex2 <- out.ex2$res
res.fmt.ex2 <- out.ex2$res.fmt
res.fmt.ex2
```

```
##      Method/Prior Parameter   Mean    SD Median            95%CI
## 1     Frequentist       muB -0.616 0.231     NA (-1.079, -0.174)
## 2     Frequentist       muC -0.342 0.128     NA (-0.598, -0.094)
## 3     Frequentist     delta -0.274 0.264     NA  (-0.791, 0.244)
## 4  Normal (vague)       muB -0.616 0.232 -0.615 (-1.073, -0.160)
## 5  Normal (vague)       muC -0.342 0.128 -0.343 (-0.595, -0.091)
## 6  Normal (vague)     delta -0.274 0.265 -0.273  (-0.792, 0.245)
## 7        Discrete       muB -0.617 0.231 -0.615 (-1.071, -0.169)
## 8        Discrete       muC -0.343 0.128 -0.343 (-0.593, -0.092)
## 9        Discrete     delta -0.274 0.264 -0.300  (-0.800, 0.200)
## 10     Spike/Slab       muB -0.416 0.127 -0.412 (-0.674, -0.183)
## 11     Spike/Slab       muC -0.404 0.114 -0.405 (-0.626, -0.179)
## 12     Spike/Slab     delta -0.012 0.078 -0.001  (-0.207, 0.020)
## 13  Normal (info)       muB -0.571 0.195 -0.571 (-0.951, -0.191)
## 14  Normal (info)       muC -0.355 0.123 -0.354 (-0.598, -0.117)
## 15  Normal (info)     delta -0.216 0.207 -0.215  (-0.619, 0.190)
```

### 2. Study Design

We explore a new trial design which is similar to the METEOR example. The entire new study population is \(A=B \cup C\) with the proportion \(\pi\) of B patients. We determine probability of the trial success for scenarios \(\pi=\) 1/3, 1/2 and 2/3.

```
npts <- 900
eventrateB0 <- 0.8
eventrateB1 <- 0.6
eventrateC0 <- 0.7
eventrateC1 <- 0.5
muC.prior.mean <- 0
muC.prior.se <- 10

propB <- c(1/3, 1/2, 2/3)
propB.char <- as.character(fractions(c(1/3, 1/2, 2/3)))
pop <- c("A", "B", "C")

out.design.info <- fit.design(
  estB = estB, estC = estC, 
  sB = sB, sC = sC, 
  prior.mean = delta.prior.mean.info, 
  prior.se = delta.prior.se.info, 
  muC.prior.mean = 0, muC.prior.se = 10, 
  npts = npts, propB = propB, 
  ranrB = 1, ranrC = 1,
  eventrateB0 = eventrateB0, 
  eventrateB1 = eventrateB1,
  eventrateC0 = eventrateC0, 
  eventrateC1 = eventrateC1
)
```

```
## Compiling model graph
##    Resolving undeclared variables
##    Allocating nodes
## Graph information:
##    Observed stochastic nodes: 2
##    Unobserved stochastic nodes: 8
##    Total graph size: 131
## 
## Initializing model
```

```
sig.mat <- out.design.info$results[,grep("sig", colnames(out.design.info$results))]
colnames(sig.mat) <- gsub("\\[|\\]", "", colnames(sig.mat))
sig.mat <- data.frame(sig.mat)

# create a summary table for power
sum.design <- expand.grid(Population = pop, PropB = propB.char) %>% 
  arrange_all()
sum.design$Power <- sprintf(colMeans(sig.mat), fmt = '%#.3f')
sum.design
```

```
##   Population PropB Power
## 1          A   1/3 0.969
## 2          A   1/2 0.978
## 3          A   2/3 0.981
## 4          B   1/3 0.894
## 5          B   1/2 0.941
## 6          B   2/3 0.959
## 7          C   1/3 0.825
## 8          C   1/2 0.757
## 9          C   2/3 0.635
```

We also plot the posterior densities of the three Z statistics for various choices of the proportion of the B population.

```
Zmat <- out.design.info$results[, grep("Z", colnames(out.design.info$results))]
colnames(Zmat) <- gsub("\\[|\\]", "", colnames(Zmat))
Zmat <- data.frame(Zmat, check.names = F)

# create a dataframe for plots
data.3z <- data.frame(
  ZA = c(Zmat$ZA1, Zmat$ZA2, Zmat$ZA3),
  ZB = c(Zmat$ZB1, Zmat$ZB2, Zmat$ZB3),
  ZC = c(Zmat$ZC1, Zmat$ZC2, Zmat$ZC3),
  PropB = paste0("PropB=", c("1/3", "1/2", "2/3"))
)
data.3z$PropB <- factor(data.3z$PropB, levels = paste0("PropB=", c("1/3", "1/2", "2/3")))

# create a density ggplot all PropB scns
# convert data.3z from wide to long
data.long <- data.3z %>% 
  pivot_longer(
    cols = ZA : ZC, 
    names_to = "Zgroup",
    values_to = "Z"
  ) %>% mutate(group = gsub("Z", "", Zgroup))

cutoff <- -1.96
plot.title <- "Posterior Predictive Distributions"

# basic density plot in facet.grid to produce plotdata for plot with shaded area
denplot <- ggplot(data.long, aes(x = Z)) + 
  geom_density() +  
  geom_vline(xintercept = cutoff, color = "red", linetype = "dashed") + 
  theme_bw() + ylab("Density") + 
  facet_grid(vars(PropB), vars(Zgroup), scales = "free") + 
  ggtitle(plot.title) + 
  theme(plot.title = element_text(hjust = 0.5))

# extract density data and shade area under density curves
ggdata <- ggplot_build(denplot)
dendata <- data.frame(
  Z = ggdata$data[[1]]$x,
  y = ggdata$data[[1]]$density,
  panel = ggdata$data[[1]]$PANEL,
  area = ifelse(ggdata$data[[1]]$x <= -1.96, "sig", "non-sig")
)

Zgroup.labels <- c("Z[A]", "Z[B]", "Z[C]")
PropB.labels <- c('pi[B]*"=1/3"', 'pi[B]*"=1/2"', 'pi[B]*"=2/3"')
dendata$Zgroup <- ifelse(dendata$panel %in% c(1, 4, 7), "ZA", ifelse(dendata$panel %in% c(2, 5, 8), "ZB", "ZC"))
dendata$PropB <- ifelse(dendata$panel %in% c(1:3), "PropB=1/3", ifelse(dendata$panel %in% c(4:6), "PropB=1/2", "PropB=2/3"))
dendata$Zgroup <- factor(dendata$Zgroup, levels = c("ZA","ZB","ZC"), labels = Zgroup.labels)
dendata$PropB <- factor(dendata$PropB, levels = paste0("PropB=", c("1/3", "1/2", "2/3")), labels = PropB.labels)

# create cutoff anno text
anno.cutoff <- data.frame(
  Z = cutoff, 
  y = 0.25, 
  label = as.character(cutoff), 
  area = NA,
  PropB = factor("PropB=1/3", levels = paste0("PropB=", c("1/3", "1/2", "2/3")), labels = PropB.labels)
)

# create power anno text
anno.power <- sum.design %>%
  mutate(
    Zgroup = paste0("Z", Population),
    PropB = paste0("PropB=", PropB),
    label = paste0("BPP=", Power),
    Z = -12,
    y = 0.25,
    area = NA
  )
anno.power$Zgroup <- factor(anno.power$Zgroup, levels = unique(anno.power$Zgroup), labels = Zgroup.labels)
anno.power$PropB <- factor(anno.power$PropB, levels = unique(anno.power$PropB), labels = PropB.labels)

denplot.fill <- ggplot(data = dendata, aes(x = Z, ymin = 0, ymax = y, fill = area))+
  geom_ribbon(aes(alpha = area)) +
  geom_line(aes(y = y)) + 
  geom_vline(xintercept = cutoff, color = "red", linetype = "dashed") + 
  theme_bw() + ylab("Density") +
  geom_text(data = anno.power, mapping = aes(x = Z, y = y, label = label), size = 3, color = "black") + 
  scale_alpha_manual(values = c("sig" = 0.1, "nonsig" = 1)) + 
  theme(legend.position = "none") + 
  facet_grid(vars(PropB), vars(Zgroup),
             labeller = label_parsed) + 
  geom_text(data = anno.cutoff, mapping = aes(x = Z, y = y, label = as.character(cutoff)), hjust = -0.12, size = 3, color = "red") +
  ggtitle(plot.title) +
  theme(plot.title = element_text(hjust = 0.5))
denplot.fill
```

```
ggsave("denplot.png", plot = denplot.fill, width = 7, height = 5, dpi = 500)
```

### 3. Additional priors

We extend our methodology by integrating the following additional advanced priors:

- Spike/slab for \(\delta\) with a range of variances for the slab
- Power prior for \(\mu\_C\) with a variety of k
- Truncated normal prior for \(\mu\_C\) with \(\mu\_C\) at least as good as \(\mbox{log}(0.8)\)
- Vague joint prior for \(\mu\_B\) and \(\mu\_C\)
- Exotic joint prior for \(\mu\_B\) and \(\mu\_C\)

Further elaboration on these details for both examples can be found in the subsequent sections.

#### 3.1 STAMPEDE

##### 3.1.1 Spike and slab prior for \(\delta\)

Run the spike and slab prior with additional variances for the slab \(\tau^2=1, 0.09\) to show how the results are sensitive to this.

```
vSlab.vec <- c(1,0.09)
vSlab.char <- as.character(vSlab.vec)
out.vslab <- vector("list", length = length(vSlab.vec))

for(i in 1:length(vSlab.vec)){
  out.vslab[[i]] <- fit.ss(
    estB = est.ex1$estB, 
    estC = est.ex1$estC,
    sB = est.ex1$sB, 
    sC = est.ex1$sC, 
    vSlab = vSlab.vec[i]
  )
}
```

```
## Compiling model graph
##    Resolving undeclared variables
##    Allocating nodes
## Graph information:
##    Observed stochastic nodes: 2
##    Unobserved stochastic nodes: 5
##    Total graph size: 23
## 
## Initializing model
## 
## Compiling model graph
##    Resolving undeclared variables
##    Allocating nodes
## Graph information:
##    Observed stochastic nodes: 2
##    Unobserved stochastic nodes: 5
##    Total graph size: 23
## 
## Initializing model
```

```
vars.ss <- c("Islab", "muB", "muC", "delta")
methods.ss <- paste0("vSpike=0.0001, vSlab=", vSlab.char)
names(out.vslab) <- methods.ss
sum.vslab.ex1 <- gen.sum(out.list = out.vslab, ndigit = 3, params = vars.ss)
res.vslab.ex1 <- sum.vslab.ex1$res
res.fmt.vslab.ex1 <- sum.vslab.ex1$res.fmt
res.fmt.vslab.ex1
```

```
##                Method/Prior Parameter   Mean    SD Median            95%CI
## 1    vSpike=0.0001, vSlab=1     Islab  0.254 0.435  0.000   (0.000, 1.000)
## 2    vSpike=0.0001, vSlab=1       muB -0.417 0.157 -0.438 (-0.665, -0.011)
## 3    vSpike=0.0001, vSlab=1       muC -0.466 0.102 -0.465 (-0.669, -0.270)
## 4    vSpike=0.0001, vSlab=1     delta  0.049 0.150  0.002  (-0.123, 0.510)
## 5 vSpike=0.0001, vSlab=0.09     Islab  0.437 0.496  0.000   (0.000, 1.000)
## 6 vSpike=0.0001, vSlab=0.09       muB -0.414 0.151 -0.428 (-0.678, -0.070)
## 7 vSpike=0.0001, vSlab=0.09       muC -0.466 0.101 -0.466 (-0.664, -0.267)
## 8 vSpike=0.0001, vSlab=0.09     delta  0.052 0.141  0.005  (-0.186, 0.424)
```

##### 3.1.2 Power prior for \(\mu\_C\) with a variety of k

Consider a power prior with a scalar parameter k for \(\mu\_C\), \(0<k<1\), to downweight the external \(\mu\_C\) information in the STAMPEDE example.

Let k=0.75, 0.5, 0.25, and the updated prior \(\mu\_C \sim N(-0.288, 0.108^2/k)\).

```
k.vec <- c(0.75, 0.5, 0.25)
out.pp <- vector("list", length(k.vec))
for(i in 1:length(k.vec)){
  out.pp[[i]] <- fit.cont(
    estB = est.ex1$estB, 
    estC = est.ex1$estC, 
    sB = est.ex1$estB, 
    sC = est.ex1$sC,
    muC.prior.mean = muC.prior.mean.info, 
    muC.prior.se = muC.prior.se.info, 
    k.muC = k.vec[i]
  )
}
```

```
## Compiling model graph
##    Resolving undeclared variables
##    Allocating nodes
## Graph information:
##    Observed stochastic nodes: 2
##    Unobserved stochastic nodes: 2
##    Total graph size: 22
## 
## Initializing model
## 
## Compiling model graph
##    Resolving undeclared variables
##    Allocating nodes
## Graph information:
##    Observed stochastic nodes: 2
##    Unobserved stochastic nodes: 2
##    Total graph size: 22
## 
## Initializing model
## 
## Compiling model graph
##    Resolving undeclared variables
##    Allocating nodes
## Graph information:
##    Observed stochastic nodes: 2
##    Unobserved stochastic nodes: 2
##    Total graph size: 22
## 
## Initializing model
```

```
methods.pp <- paste0("power prior for muC, k=", k.vec)
names(out.pp) <- methods.pp
sum.pp.ex1 <- gen.sum(out.list = out.pp, ndigit = 3, params = vars)
res.pp.ex1 <- sum.pp.ex1$res
res.fmt.pp.ex1 <- sum.pp.ex1$res.fmt
res.fmt.pp.ex1
```

```
##                  Method/Prior Parameter   Mean    SD Median            95%CI
## 1 power prior for muC, k=0.75       muB -0.287 0.287 -0.287  (-0.854, 0.276)
## 2 power prior for muC, k=0.75       muC -0.405 0.082 -0.405 (-0.565, -0.244)
## 3 power prior for muC, k=0.75     delta  0.117 0.298  0.117  (-0.472, 0.700)
## 4  power prior for muC, k=0.5       muB -0.289 0.288 -0.288  (-0.851, 0.272)
## 5  power prior for muC, k=0.5       muC -0.425 0.088 -0.425 (-0.597, -0.253)
## 6  power prior for muC, k=0.5     delta  0.135 0.301  0.135  (-0.455, 0.725)
## 7 power prior for muC, k=0.25       muB -0.287 0.288 -0.288  (-0.853, 0.281)
## 8 power prior for muC, k=0.25       muC -0.453 0.096 -0.453 (-0.641, -0.262)
## 9 power prior for muC, k=0.25     delta  0.166 0.304  0.166  (-0.428, 0.770)
```

##### 3.1.3 More informative prior (truncated prior) for \(\mu\_C\)

We assume \(\mu\_C \leq \mbox{log}(0.8)\), i.e., we are confident or “know” that the HR in subgroup C is equal to or superior to 0.8. We implement a truncated prior for \(\mu\_C\) to account for this upper bound constraint on its distribution.

Note that, the function T() in JAGS is used to truncate the normal distribution function of \(\mu\_C\). However, it can’t be directly called in R script, instead, the model should be written and saved in a separate text file. This model file is then referenced in the R code.

```
jags.data <- list(
  estB = est.ex1$estB,
  estC = est.ex1$estC,
  sB = est.ex1$sB,
  sC = est.ex1$sC,
  prior.mean = 0,
  prior.se = 10,
  muC.prior.mean = 0,
  muC.prior.se = 10
)

jags.init <- function() { list(muC = -0.5) }  ### initial muC can't be 0 when using trunc dist
jags.parm <- c("delta", "muB", "muC")

jags.fit <- jags(
  data = jags.data, 
  inits = jags.init, 
  parameters.to.save = jags.parm, 
  model.file = "model_truncprior.txt",  
  n.chains = 2, n.iter = 50000, n.burnin = 20000, n.thin = 2,
  DIC = TRUE, progress.bar = "none"
)
```

```
## Compiling model graph
##    Resolving undeclared variables
##    Allocating nodes
## Graph information:
##    Observed stochastic nodes: 2
##    Unobserved stochastic nodes: 2
##    Total graph size: 20
## 
## Initializing model
```

```
jags.mcmc <- as.mcmc(jags.fit)
jags.mcmc <- rbind(jags.mcmc[[1]], jags.mcmc[[2]])

out.trunc <- vector("list", length = 1)
out.trunc[[1]]$results <- jags.mcmc
out.trunc[[1]]$sum <- jags.fit

methods.trunc <- "truncated muC prior"
names(out.trunc) <- methods.trunc
sum.trunc.ex1 <- gen.sum(out.list = out.trunc, ndigit = 3, params = vars)
res.trunc.ex1 <- sum.trunc.ex1$res
res.fmt.trunc.ex1 <- sum.trunc.ex1$res.fmt
res.fmt.trunc.ex1
```

```
##          Method/Prior Parameter   Mean    SD Median            95%CI
## 1 truncated muC prior       muB -0.288 0.227 -0.288  (-0.735, 0.157)
## 2 truncated muC prior       muC -0.497 0.106 -0.495 (-0.710, -0.293)
## 3 truncated muC prior     delta  0.209 0.251  0.208  (-0.279, 0.699)
```

##### 3.1.4 Vague joint prior for \(\mu\_B\) and \(\mu\_C\)

Use a joint prior for \(\mu\_B\) and \(\mu\_C\), with correlation=0.5, so that \(\mu\_B\), \(\mu\_B\), \(\delta\) all have the same marginal vague priors.

```
rho <- 0.5
muBC.mean.prior <- c(0, 0)
muB.se.prior <- 10
muC.se.prior <- 10

out.joint <- fit.joint(
  estB = est.ex1$estB, 
  estC = est.ex1$estC, 
  sB = est.ex1$sB, 
  sC = est.ex1$sC, 
  muBC.mean.prior = muBC.mean.prior, 
  muB.se.prior = muB.se.prior, 
  muC.se.prior = muC.se.prior, 
  rho = rho
)
```

```
## Compiling model graph
##    Resolving undeclared variables
##    Allocating nodes
## Graph information:
##    Observed stochastic nodes: 2
##    Unobserved stochastic nodes: 1
##    Total graph size: 24
## 
## Initializing model
```

```
vars.vjoint <- c("muBC[1]", "muBC[2]", "delta")
methods.vjoint <- "vague joint prior for muB and muC"

out.vjoint <- vector("list", length = 1)
out.vjoint[[1]] <- out.joint
names(out.vjoint) <- methods.vjoint

sum.vjoint.ex1 <- gen.sum(out.list = out.vjoint, ndigit = 3, params = vars.vjoint)
res.vjoint.ex1 <- sum.vjoint.ex1$res
res.vjoint.ex1$Parameter <- vars
res.fmt.vjoint.ex1 <- sum.vjoint.ex1$res.fmt
res.fmt.vjoint.ex1$Parameter <- vars
res.fmt.vjoint.ex1
```

```
##                        Method/Prior Parameter   Mean    SD Median
## 1 vague joint prior for muB and muC       muB -0.286 0.230 -0.285
## 2 vague joint prior for muB and muC       muC -0.495 0.107 -0.496
## 3 vague joint prior for muB and muC     delta  0.209 0.254  0.209
##              95%CI
## 1  (-0.739, 0.163)
## 2 (-0.706, -0.283)
## 3  (-0.298, 0.706)
```

##### 3.1.5 Exotic joint prior for \(\mu\_B\) and \(\mu\_C\)

Informative joint prior distributions are elicited using a discrete set of combinations for \(\mu\_B\) and \(\mu\_C\). To guarantee the stability of our prior calculations, we explore three distinct sets of starting values. This approach yields consistent results with all convergence codes returning as zero, which indicates successful optimizations. The optimal values for the priors are then identified by minimizing the ‘fn’ value, which is the output from the optim() function.

```
# use a discrete set of combinations of mu_B and mu_C to elicit informative prior distributions
priors <- c(0.0030, 0.0090, 0.0150, 0.0000, 0.0000, 0.0000, 
            0.0025, 0.0315, 0.0300, 0.0175, 0.0000, 0.0000, 
            0.0020, 0.0225, 0.1050, 0.0525, 0.0100, 0.0005, 
            0.0015, 0.0135, 0.0900, 0.1400, 0.0400, 0.0020, 
            0.0005, 0.0090, 0.0450, 0.1050, 0.0900, 0.0050, 
            0.0005, 0.0045, 0.0150, 0.0350, 0.0600, 0.0425)
prior.grid <- matrix(priors, nrow = 6)

effect.sizes.bounds <- log(c(0.55, 0.65, 0.75, 0.85, 0.95))
typical.effect.sizes <- log(c(0.5, 0.6, 0.7, 0.8, 0.9, 1))

# compute marginal distributions of muB and muC
marginal.muB <- colSums(prior.grid)
marginal.muC <- rowSums(prior.grid)

# compute prior means
prior.mean.info.muB <- sum(marginal.muB * typical.effect.sizes)
prior.mean.info.muC <- sum(marginal.muC * typical.effect.sizes)

# compute prior standard deviations
prior.sd.info.muB <- sqrt(sum(marginal.muB * (typical.effect.sizes^2)) - prior.mean.info.muB^2)
prior.sd.info.muC <- sqrt(sum(marginal.muC * (typical.effect.sizes^2)) - prior.mean.info.muC^2)

# compute prior E[muB, muC] using matrix multiplication for efficiency
prior.exp.muB.muC <- sum(prior.grid * (typical.effect.sizes %*% t(typical.effect.sizes)))

# compute prior covariance and correlation
prior.covariance <- prior.exp.muB.muC - prior.mean.info.muB * prior.mean.info.muC
prior.correlation <- prior.covariance / (prior.sd.info.muC * prior.sd.info.muB)

# fit model
out.joint.normal.informative <- fit.joint(
  estB = est.ex1$estB, 
  estC = est.ex1$estC, 
  sB = est.ex1$sB, 
  sC = est.ex1$sC, 
  muBC.mean.prior = c(prior.mean.info.muB, prior.mean.info.muC), 
  muB.se.prior = prior.sd.info.muB, 
  muC.se.prior = prior.sd.info.muC, 
  rho = prior.correlation
)
```

```
## Compiling model graph
##    Resolving undeclared variables
##    Allocating nodes
## Graph information:
##    Observed stochastic nodes: 2
##    Unobserved stochastic nodes: 1
##    Total graph size: 25
## 
## Initializing model
```

```
out.joint.normal.informative$sum
```

```
## Inference for Bugs model at "C:/Users/kpvp028/AppData/Local/Temp/RtmpIBhp0H/model65301f703fac.txt", fit using jags,
##  2 chains, each with 50000 iterations (first 20000 discarded), n.thin = 2
##  n.sims = 30000 iterations saved
##          mu.vect sd.vect   2.5%    25%    50%    75%  97.5%  Rhat n.eff
## delta      0.092   0.124 -0.151  0.008  0.093  0.175  0.338 1.001 11000
## muBC[1]   -0.310   0.125 -0.552 -0.394 -0.311 -0.227 -0.063 1.001  6000
## muBC[2]   -0.402   0.084 -0.564 -0.459 -0.402 -0.346 -0.238 1.001 30000
## deviance  -2.094   1.610 -3.663 -3.237 -2.587 -1.474  2.283 1.001 30000
## 
## For each parameter, n.eff is a crude measure of effective sample size,
## and Rhat is the potential scale reduction factor (at convergence, Rhat=1).
## 
## DIC info (using the rule, pD = var(deviance)/2)
## pD = 1.3 and DIC = -0.8
## DIC is an estimate of expected predictive error (lower deviance is better).
```

```
vars.joint <- c("muBC[1]", "muBC[2]", "delta")
methods.joint <- "informative joint normal prior for muB and muC"

out.joint <- vector("list", length = 1)
out.joint[[1]] <- out.joint.normal.informative
names(out.joint) <- methods.joint

sum.joint.ex1 <- gen.sum(out.list = out.joint, ndigit = 3, params = vars.joint)
res.joint.ex1 <- sum.joint.ex1$res
res.joint.ex1$Parameter <- vars
res.fmt.joint.ex1 <- sum.joint.ex1$res.fmt
res.fmt.joint.ex1$Parameter <- vars
res.fmt.joint.ex1
```

```
##                                     Method/Prior Parameter   Mean    SD Median
## 1 informative joint normal prior for muB and muC       muB -0.310 0.125 -0.311
## 2 informative joint normal prior for muB and muC       muC -0.402 0.084 -0.402
## 3 informative joint normal prior for muB and muC     delta  0.092 0.124  0.093
##              95%CI
## 1 (-0.552, -0.063)
## 2 (-0.564, -0.238)
## 3  (-0.151, 0.338)
```

```
# derive a correlated joint prior distribution using expert elicitation
# try starting values with all zeros
results1 <- optim(
  c(0, 0, 0, 0, 0), 
  prior.calculate,
  prior.probs = prior.grid, 
  effect.sizes.bounds = effect.sizes.bounds,
  typical.effect.sizes = typical.effect.sizes,
  control = list(maxit = 100000), 
  method = "BFGS"
)
results1
```

```
## $par
## [1] -0.25203827 -4.05698795  0.81604592 -5.80644144 -0.04475995
## 
## $value
## [1] 0.001055884
## 
## $counts
## function gradient 
##      460      389 
## 
## $convergence
## [1] 0
## 
## $message
## NULL
```

```
# other starting values
results2 <- optim(
  c(log(0.7), 0.5, log(0.7), 0.5, 0), 
  prior.calculate,
  prior.probs = prior.grid, 
  effect.sizes.bounds = effect.sizes.bounds,
  typical.effect.sizes = typical.effect.sizes,
  control = list(maxit = 100000), 
  method = "BFGS"
)
results2
```

```
## $par
## [1] -0.25217289 -4.05762197  0.81598109 -5.83917888 -0.04518323
## 
## $value
## [1] 0.001055426
## 
## $counts
## function gradient 
##      426      327 
## 
## $convergence
## [1] 0
## 
## $message
## NULL
```

```
results3 <- optim(
  c(log(0.5), -2, log(0.5), -3, 1), 
  prior.calculate,
  prior.probs = prior.grid, 
  effect.sizes.bounds = effect.sizes.bounds,
  typical.effect.sizes = typical.effect.sizes,
  control = list(maxit = 100000), 
  method = "BFGS"
)
results3
```

```
## $par
## [1] -0.25208981 -4.05768083  0.81592552 -5.82064632 -0.04494755
## 
## $value
## [1] 0.001055638
## 
## $counts
## function gradient 
##      687      577 
## 
## $convergence
## [1] 0
## 
## $message
## NULL
```

```
# consistent results for 3 different starting values, and convergence codes of 0. 
# find the optimal set of starting values by minimizing the fn value.
results.all <- list(results1, results2, results3)
results <- results.all[[which.min(c(results1$value, results2$value, results3$value))]]

out.rectified <- fit.rectified(
  estB = est.ex1$estB, 
  estC = est.ex1$estC,
  sB = est.ex1$sB, 
  sC = est.ex1$sC, 
  muC.prior.mean = results$par[1], 
  muC.prior.var = exp(results$par[2]), 
  muB.cond.prior.mean.scale = results$par[3], 
  muB.cond.prior.var.shift = exp(results$par[4]), 
  muB.cond.prior.var.scale = results$par[5]
)
```

```
## Compiling model graph
##    Resolving undeclared variables
##    Allocating nodes
## Graph information:
##    Observed stochastic nodes: 2
##    Unobserved stochastic nodes: 2
##    Total graph size: 26
## 
## Initializing model
```

```
methods.ejoint <- "exotic novel joint prior for muB and muC"
out.ejoint <- vector("list", length = 1)
out.ejoint[[1]] <- out.rectified
names(out.ejoint) <- methods.ejoint

sum.ejoint.ex1 <- gen.sum(out.list = out.ejoint, ndigit = 3, params = vars)
res.ejoint.ex1 <- sum.ejoint.ex1$res
res.fmt.ejoint.ex1 <- sum.ejoint.ex1$res.fmt
res.fmt.ejoint.ex1
```

```
##                               Method/Prior Parameter   Mean    SD Median
## 1 exotic novel joint prior for muB and muC       muB -0.307 0.130 -0.304
## 2 exotic novel joint prior for muB and muC       muC -0.391 0.081 -0.391
## 3 exotic novel joint prior for muB and muC     delta  0.084 0.126  0.082
##              95%CI
## 1 (-0.570, -0.056)
## 2 (-0.552, -0.232)
## 3  (-0.159, 0.336)
```

```
# ggplot2 2d density contour
# https://ggplot2.tidyverse.org/reference/geom_density_2d.html

out.rectified.prior <- fit.rectified.no.data(
  muC.prior.mean = results$par[1], 
  muC.prior.var = exp(results$par[2]), 
  muB.cond.prior.mean.scale = results$par[3], 
  muB.cond.prior.var.shift = exp(results$par[4]), 
  muB.cond.prior.var.scale = results$par[5]
)
```

```
## Compiling model graph
##    Resolving undeclared variables
##    Allocating nodes
## Graph information:
##    Observed stochastic nodes: 0
##    Unobserved stochastic nodes: 2
##    Total graph size: 18
## 
## Initializing model
```

```
priordata <- data.frame(out.rectified.prior$results) %>%
    mutate(muB.prior = muB, muC.prior = muC)
priorplot <- ggplot(data = priordata, aes(x = muB.prior, y = muC.prior)) +
  xlim(-0.75, 0) + ylim(-0.75, 0) + 
  geom_density_2d_filled() +
  ggtitle(expression(Joint~Novel~Prior~Distribution~of~mu[B]~and~mu[C])) + 
  xlab(expression(mu[B])) +
  ylab(expression(mu[C])) + 
  theme_bw() + 
  theme(plot.title = element_text(hjust = 0.5), legend.position = "none")
priorplot
```

```
# get posterior muB and muC
postdata <- data.frame(out.rectified$results) %>% 
  mutate(muB.posterior = muB, muC.posterior = muC)
postplot <- ggplot(data = postdata, aes(x = muB.posterior, y = muC.posterior)) + 
  xlim(-0.75, 0) + ylim(-0.75, 0) + 
  geom_density_2d_filled() +
  ggtitle(expression(Joint~Posterior~Distribution~of~mu[B]~and~mu[C])) + 
  xlab(expression(mu[B])) +
  ylab(expression(mu[C])) + 
  theme_bw() + 
  theme(plot.title = element_text(hjust = 0.5), legend.position = "none")
postplot
```

```
ggsave("priorplot.png", plot = priorplot, width = 7, height = 5, dpi = 500)
ggsave("postplot.png", plot = postplot, width = 7, height = 5, dpi = 500)
```

##### Summary

Combine all the results in one table along with a forest plot.

```
res.add.ex1 <- rbind(res.vslab.ex1, res.pp.ex1, res.trunc.ex1, res.vjoint.ex1)
res.add.ex1 <- subset(res.add.ex1, Parameter != "Islab")

# create a plotdata frame for forest plot
res.plot <- rbind(subset(res.ex1, `Method/Prior` == "Normal (vague)"),
                  subset(res.add.ex1, `Method/Prior` == "vague joint prior for muB and muC"), 
                  subset(res.add.ex1, `Method/Prior` == "truncated muC prior"),
                  subset(res.add.ex1, `Method/Prior` == "power prior for muC, k=0.25"),
                  subset(res.add.ex1, `Method/Prior` == "power prior for muC, k=0.5"),
                  subset(res.add.ex1, `Method/Prior` == "power prior for muC, k=0.75"),
                  subset(res.ex1, `Method/Prior` == "Normal (info)"),
                  subset(res.add.ex1, `Method/Prior` == "vSpike=0.0001, vSlab=0.09"),
                  subset(res.add.ex1, `Method/Prior` == "vSpike=0.0001, vSlab=1"),
                  subset(res.ex1, `Method/Prior` == "Spike/Slab"))
prior.labels.greek <- c("Normal (vague)", "Joint (vague)", "Normal truncated",
                        "Normal (info), k=0.25", "Normal (info), k=0.5", "Normal (info), k=0.75", "Normal (info), k=1",
                        "Spike/Slab, \u03c4=0.3", "Spike/Slab, \u03c4=1", "Spike/Slab, \u03c4=10")

res.plot$`Method/Prior` <- rep(prior.labels.greek, each = 3)
plotdata <- data.frame(res.plot, check.names = F)

forestplot.ex1 <- gen.forestplot(plotdata = plotdata)
forestplot.ex1
```

```
ggsave("forestplot_ex1.png", plot = forestplot.ex1, width = 8, height = 8, dpi = 500)
```

#### 3.2 METEOR

##### 3.2.1 Spike and slab prior for \(\delta\)

Run the spike and slab prior with a range of variances for the slab \(\tau^2=1, 0.09\) to show how the results are sensitive to this.

```
vSlab.vec <- c(1,0.09)
vSlab.char <- as.character(vSlab.vec)
out.vslab <- vector("list", length = length(vSlab.vec))

for(i in 1:length(vSlab.vec)){
  out.vslab[[i]] <- fit.ss(
    estB = est.ex2$estB, 
    estC = est.ex2$estC,
    sB = est.ex2$sB, 
    sC = est.ex2$sC, 
    vSlab = vSlab.vec[i]
  )
}
```

```
## Compiling model graph
##    Resolving undeclared variables
##    Allocating nodes
## Graph information:
##    Observed stochastic nodes: 2
##    Unobserved stochastic nodes: 5
##    Total graph size: 23
## 
## Initializing model
## 
## Compiling model graph
##    Resolving undeclared variables
##    Allocating nodes
## Graph information:
##    Observed stochastic nodes: 2
##    Unobserved stochastic nodes: 5
##    Total graph size: 23
## 
## Initializing model
```

```
vars.ss <- c("Islab", "muB", "muC", "delta")
methods.ss <- paste0("vSpike=0.0001, vSlab=", vSlab.char)
names(out.vslab) <- methods.ss
sum.vslab.ex2 <- gen.sum(out.list = out.vslab, ndigit = 3, params = vars.ss)
res.vslab.ex2 <- sum.vslab.ex2$res
res.fmt.vslab.ex2 <- sum.vslab.ex2$res.fmt
res.fmt.vslab.ex2
```

```
##                Method/Prior Parameter   Mean    SD Median            95%CI
## 1    vSpike=0.0001, vSlab=1     Islab  0.302 0.459  0.000   (0.000, 1.000)
## 2    vSpike=0.0001, vSlab=1       muB -0.467 0.180 -0.440 (-0.915, -0.181)
## 3    vSpike=0.0001, vSlab=1       muC -0.390 0.120 -0.391 (-0.620, -0.150)
## 4    vSpike=0.0001, vSlab=1     delta -0.078 0.183 -0.004  (-0.610, 0.097)
## 5 vSpike=0.0001, vSlab=0.09     Islab  0.470 0.499  0.000   (0.000, 1.000)
## 6 vSpike=0.0001, vSlab=0.09       muB -0.463 0.165 -0.446 (-0.832, -0.177)
## 7 vSpike=0.0001, vSlab=0.09       muC -0.390 0.118 -0.391 (-0.621, -0.156)
## 8 vSpike=0.0001, vSlab=0.09     delta -0.073 0.156 -0.007  (-0.475, 0.166)
```

##### 3.2.2 Power prior for \(\delta\)

Consider a power prior with a scalar parameter k, \(0<k<1\), to downweight the external \(\delta\) information in the METEOR example.

Let k=0.75, 0.5, 0.25, and the updated prior \(\delta \sim N(-0.122, 0.334^2/k)\).

```
k.vec <- c(0.75, 0.5, 0.25)
out.pp <- vector("list", length(k.vec))
for(i in 1:length(k.vec)){
  out.pp[[i]] <- fit.cont(
    estB = est.ex2$estB, 
    estC = est.ex2$estC, 
    sB = est.ex2$sB, 
    sC = est.ex2$sC, 
    prior.mean = delta.prior.mean.info, 
    prior.se = delta.prior.se.info, 
    k.delta = k.vec[i]
  )
}
```

```
## Compiling model graph
##    Resolving undeclared variables
##    Allocating nodes
## Graph information:
##    Observed stochastic nodes: 2
##    Unobserved stochastic nodes: 2
##    Total graph size: 22
## 
## Initializing model
## 
## Compiling model graph
##    Resolving undeclared variables
##    Allocating nodes
## Graph information:
##    Observed stochastic nodes: 2
##    Unobserved stochastic nodes: 2
##    Total graph size: 22
## 
## Initializing model
## 
## Compiling model graph
##    Resolving undeclared variables
##    Allocating nodes
## Graph information:
##    Observed stochastic nodes: 2
##    Unobserved stochastic nodes: 2
##    Total graph size: 22
## 
## Initializing model
```

```
methods.pp <- paste0("power prior for delta, k=", k.vec)
names(out.pp) <- methods.pp
sum.pp.ex2 <- gen.sum(out.list = out.pp, ndigit = 3, params = vars)
res.pp.ex2 <- sum.pp.ex2$res
res.fmt.pp.ex2 <- sum.pp.ex2$res.fmt
res.fmt.pp.ex2
```

```
##                    Method/Prior Parameter   Mean    SD Median            95%CI
## 1 power prior for delta, k=0.75       muB -0.578 0.201 -0.578 (-0.971, -0.183)
## 2 power prior for delta, k=0.75       muC -0.353 0.124 -0.353 (-0.596, -0.110)
## 3 power prior for delta, k=0.75     delta -0.225 0.219 -0.226  (-0.652, 0.204)
## 4  power prior for delta, k=0.5       muB -0.588 0.208 -0.588 (-0.993, -0.179)
## 5  power prior for delta, k=0.5       muC -0.351 0.124 -0.351 (-0.595, -0.108)
## 6  power prior for delta, k=0.5     delta -0.237 0.229 -0.238  (-0.682, 0.214)
## 7 power prior for delta, k=0.25       muB -0.599 0.219 -0.599 (-1.030, -0.168)
## 8 power prior for delta, k=0.25       muC -0.347 0.126 -0.347 (-0.591, -0.101)
## 9 power prior for delta, k=0.25     delta -0.252 0.244 -0.253  (-0.731, 0.222)
```

##### 3.2.3 More informative prior (truncated prior) for \(\mu\_C\)

We assume \(\mu\_C \leq \mbox{log}(0.8)\), i.e., we are confident or “know” that the HR in subgroup C is equal to or superior to 0.8. We implement a truncated prior for \(\mu\_C\) to account for this upper bound constraint on its distribution.

```
jags.data <- list(
  estB = est.ex2$estB,
  estC = est.ex2$estC,
  sB = est.ex2$sB,
  sC = est.ex2$sC,
  prior.mean = 0,
  prior.se = 10,
  muC.prior.mean = 0,
  muC.prior.se = 10
)

jags.init <- function() { list(muC = -0.5) }  ### initial muC can't be 0 when using trunc dist
jags.parm <- c("delta", "muB", "muC")

jags.fit <- jags(
  data = jags.data, 
  inits = jags.init, 
  parameters.to.save = jags.parm, 
  model.file = "model_truncprior.txt",  
  n.chains = 2, n.iter = 50000, n.burnin = 20000, n.thin = 2,
  DIC = TRUE, progress.bar = "none"
)
```

```
## Compiling model graph
##    Resolving undeclared variables
##    Allocating nodes
## Graph information:
##    Observed stochastic nodes: 2
##    Unobserved stochastic nodes: 2
##    Total graph size: 20
## 
## Initializing model
```

```
jags.mcmc <- as.mcmc(jags.fit)
jags.mcmc <- rbind(jags.mcmc[[1]], jags.mcmc[[2]])

out.trunc <- vector("list", length = 1)
out.trunc[[1]]$results <- jags.mcmc
out.trunc[[1]]$sum <- jags.fit

methods.trunc <- "truncated muC prior"
names(out.trunc) <- methods.trunc
sum.trunc.ex2 <- gen.sum(out.list = out.trunc, ndigit = 3, params = vars)
res.trunc.ex2 <- sum.trunc.ex2$res
res.fmt.trunc.ex2 <- sum.trunc.ex2$res.fmt
res.fmt.trunc.ex2
```

```
##          Method/Prior Parameter   Mean    SD Median            95%CI
## 1 truncated muC prior       muB -0.616 0.230 -0.616 (-1.065, -0.166)
## 2 truncated muC prior       muC -0.383 0.100 -0.372 (-0.607, -0.233)
## 3 truncated muC prior     delta -0.233 0.250 -0.234  (-0.723, 0.259)
```

##### 3.2.4 Vague joint prior for \(\mu\_B\) and \(\mu\_C\)

Use a joint prior for \(\mu\_B\) and \(\mu\_C\), with correlation=0.5, so that \(\mu\_B\), \(\mu\_B\), \(\delta\) all have the same marginal vague priors.

```
rho <- 0.5
muBC.mean.prior <- c(0,0)
muB.se.prior <- 10
muC.se.prior <- 10

out.joint <- fit.joint(
  estB = est.ex2$estB, 
  estC = est.ex2$estC, 
  sB = est.ex2$sB, 
  sC = est.ex2$sC, 
  muBC.mean.prior = muBC.mean.prior, 
  muB.se.prior = muB.se.prior, 
  muC.se.prior = muC.se.prior, 
  rho = rho
)
```

```
## Compiling model graph
##    Resolving undeclared variables
##    Allocating nodes
## Graph information:
##    Observed stochastic nodes: 2
##    Unobserved stochastic nodes: 1
##    Total graph size: 24
## 
## Initializing model
```

```
vars.vjoint <- c("muBC[1]", "muBC[2]", "delta")
methods.vjoint <- "vague joint prior for muB and muC"

out.vjoint <- vector("list", length = 1)
out.vjoint[[1]] <- out.joint
names(out.vjoint) <- methods.vjoint

sum.vjoint.ex2 <- gen.sum(out.list = out.vjoint, ndigit = 3, params = vars.vjoint)
res.vjoint.ex2 <- sum.vjoint.ex2$res
res.vjoint.ex2$Parameter <- vars
res.fmt.vjoint.ex2 <- sum.vjoint.ex2$res.fmt
res.fmt.vjoint.ex2$Parameter <- vars
res.fmt.vjoint.ex2
```

```
##                        Method/Prior Parameter   Mean    SD Median
## 1 vague joint prior for muB and muC       muB -0.618 0.231 -0.618
## 2 vague joint prior for muB and muC       muC -0.343 0.129 -0.343
## 3 vague joint prior for muB and muC     delta -0.275 0.265 -0.277
##              95%CI
## 1 (-1.066, -0.166)
## 2 (-0.593, -0.090)
## 3  (-0.793, 0.242)
```

##### Summary

We combine all the results in one table along with a forest plot.

```
res.add.ex2 <- rbind(res.vslab.ex2, res.pp.ex2, res.trunc.ex2, res.vjoint.ex2)
res.add.ex2 <- subset(res.add.ex2, Parameter != "Islab")

# create a plotdata frame for forest plot
res.plot <- rbind(subset(res.ex2, `Method/Prior` == "Normal (vague)"),
                  subset(res.add.ex2, `Method/Prior` == "vague joint prior for muB and muC"), 
                  subset(res.add.ex2, `Method/Prior` == "truncated muC prior"),
                  subset(res.add.ex2, `Method/Prior` == "power prior for delta, k=0.25"),
                  subset(res.add.ex2, `Method/Prior` == "power prior for delta, k=0.5"),
                  subset(res.add.ex2, `Method/Prior` == "power prior for delta, k=0.75"),
                  subset(res.ex2, `Method/Prior` == "Normal (info)"),
                  subset(res.add.ex2, `Method/Prior` == "vSpike=0.0001, vSlab=0.09"),
                  subset(res.add.ex2, `Method/Prior` == "vSpike=0.0001, vSlab=1"),
                  subset(res.ex2, `Method/Prior` == "Spike/Slab"))
prior.labels.greek <- c("Normal (vague)", "Joint (vague)", "Normal truncated",
                        "Normal (info), k=0.25", "Normal (info), k=0.5", "Normal (info), k=0.75", "Normal (info), k=1",
                        "Spike/Slab, \u03c4=0.3", "Spike/Slab, \u03c4=1", "Spike/Slab, \u03c4=10")

res.plot$`Method/Prior` <- rep(prior.labels.greek, each = 3)
plotdata <- data.frame(res.plot, check.names = F)

forestplot.ex2 <- gen.forestplot(plotdata = plotdata)
forestplot.ex2
```

```
ggsave("forestplot_ex2.png", plot = forestplot.ex2, width = 8, height = 8, dpi = 500)
```
